# A genome‐wide association and replication study of blood pressure in Ugandan early adolescents

**DOI:** 10.1002/mgg3.950

**Published:** 2019-08-30

**Authors:** Swaib A. Lule, Alexander J. Mentzer, Benigna Namara, Allan G. Muwenzi, Beatrice Nassanga, Dennison kizito, Helen Akurut, Lawrence Lubyayi, Josephine Tumusiime, Christopher Zziwa, Florence Akello, Deept Gurdasani, Manjinder Sandhu, Liam Smeeth, Alison M. Elliott, Emily L. Webb

**Affiliations:** ^1^ London School of Hygiene and Tropical Medicine London UK; ^2^ MRC/UVRI & LSHTM Uganda Research Unit Entebbe Uganda; ^3^ Wellcome Trust Centre for Human Genetics University of Oxford Oxford UK; ^4^ Big Data Institute, Li Ka Shing Centre for Health Information and Discovery University of Oxford Oxford UK; ^5^ Wellcome Trust Sanger Institute Cambridge UK; ^6^ Uganda Virus Research Institute Entebbe Uganda; ^7^ University of Cambridge Cambridge UK

**Keywords:** adolescents, Africa, blood pressure, genetics, replication analysis, single nucleotide polymorphisms, Uganda

## Abstract

**Background:**

Genetic association studies of blood pressure (BP) have mostly been conducted in non‐African populations. Using the Entebbe Mother and Baby Study (EMaBS), we aimed to identify genetic variants associated with BP among Ugandan adolescents.

**Methods:**

Systolic and diastolic BP were measured among 10‐ and 11‐year olds. Whole‐genome genotype data were generated using Illumina omni 2.5M arrays and untyped variants were imputed. Genome‐wide association study (GWAS) was conducted using linear mixed model regression to account for population structure. Linear regression analysis was used to assess whether variants previously associated with BP (*p* < 5.0 × 10^−8^) in published BP GWASs were replicated in our study.

**Results:**

Of the 14 million variants analyzed among 815 adolescents, none reached genome‐wide significance (*p* < 5.0×10^−8^) for association with systolic or diastolic BP. The most strongly associated variants were rs181430167 (*p* = 6.8 × 10^−7^) for systolic BP and rs12991132 (*p* = 4.0 × 10^−7^) for diastolic BP. Thirty‐three (17 single nucleotide polymorphisms (SNPs) for systolic BP, 15 SNPs for diastolic BP and one SNP for both) of 330 variants previously identified as associated with BP were replicated in this study, but none remained significant after accounting for multiple testing.

**Conclusion:**

Variants showing suggestive associations are worthy of future investigation. Replication results suggest that variants influencing adolescent BP may overlap somewhat with those already established in previous studies, largely based on adults in Western settings.

## 1 INTRODUCTION

Genome‐wide association studies (GWAS) predominantly from Caucasian and Asian populations have identified several single nucleotide polymorphisms (SNPs) associated with blood pressure (BP; Cho et al., 2009; Evangelou et al., 2018; Levy et al., 2009; Qian, Lu, Tan, Liu, & Lu, 2007; Warren et al., 2017; Xi et al., 2014). Despite having a high burden of hypertension, and opportunities for improved fine‐mapping of causative variants, including higher genetic diversity and lower linkage disequilibrium (LD; Addo, Smeeth, & Leon, 2007; Luoni et al., 2005; Noubiap et al., 2017; Tishkoff et al., 2009), African populations are underrepresented in published genetic studies of BP (Peprah, Xu, Tekola‐Ayele, & Royal, 2015). Among 38 studies that investigated genetic polymorphisms associated with hypertension in Africa‐based populations (participant numbers ranging from 65 to 1939) reviewed in (Yako et al., 2018), all adopted a candidate gene approach rather than conducting a GWAS. It remains unclear whether variants associated with BP in non‐African populations also influence BP among Africans or whether the patterns of genetic susceptibility differ markedly.

Blood pressure GWASs conducted among populations of African origin in the diaspora are rare, and often report different variants associated with BP compared to those reported in non‐African populations (Adeyemo et al., 2009; Fox et al., 2011; Franceschini et al., 2013; Kidambi et al., 2012; Liang et al., 2017). Attempts to replicate genetic findings in independent populations have returned mixed results (Kayima et al., 2017; Kidambi et al., 2012; Li et al., 2017; Xi et al., 2014). The largest African American GWAS of BP included a meta‐analysis of 29,378 individuals, and identified only one (the *SOX6* locus) of the five loci that were associated with BP in a multiethnic (African American, European and East Asian) sample of 99,382 individuals (Franceschini et al., 2013). Of the 17 SNPs most strongly associated with BP (*p* < 1 × 10^−4^) in African Americans, three SNPS were replicated (*p* < .05) in a West African sample (Adeyemo et al., 2009). Among Ugandan adults, 11 out of 27 BP related candidate SNPs (selected because of previous association with BP from BP GWASs or admixture mapping analysis) were replicated (*p* < .05) with eight of the 11 SNPs having the same effect direction as in the discovery sample (Kayima et al., 2017).

Twin studies report that over 30% of BP variability is heritable (Biron, Mongeau, & Bertrand, 1976; Williams et al., 1991) but established variants associated with BP account for only 2%–5% of BP variation (Ehret & Caulfield, 2013; Salfati, Morrison, Boerwinkle, & Chakravarti, 2015). This strongly suggests the existence of important undiscovered variants. This “missing heritability” could be due to rare or to common SNPs, all conferring small increases or decreases in expected BP (Maher, 2008; Manolio et al., 2009). There is a need for both GWAS and replication studies to further elucidate and improve the generalizability of BP genetic findings.

The role of environmental and of lifestyle factors in hypertension among Africans is well documented as reviewed previously (Addo et al., 2007; Noubiap et al., 2017). Previously, we described the role of environmental and of lifestyle factors in adolescents’ BP and the findings were reported in (Lule, Namara, Akurut, Lubyayi, et al., 2019; Lule, Namara, Akurut, Muhangi, et al., 2019). However, the contribution of genetic variants remains unknown and understudied (Yako et al., 2018). It is not clear whether genetic loci associated with hypertension among populations of non‐African origin influence susceptibility to or protection from hypertension in populations on the African continent. Independent confirmation is necessary to validate BP SNPs in different populations. We used data from the Entebbe Mother and Baby Study (EMaBS) birth cohort (Elliott et al., 2007) to conduct 1) a GWAS of systolic and diastolic BP and 2) a replication study of candidate SNPs identified in previously published BP GWASs. The GWAS aimed to identify novel BP loci unique to this population while candidate gene analysis aimed to identify variants influencing BP across different ethnic groups.

We hypothesized that genetic variants (either unique or not unique to populations in Africa) would be associated with BP among Ugandan adolescents. Individuals of African origin have different genetic makeup from individuals of non‐African ancestries (Addo et al., 2007; Luoni et al., 2005; Noubiap et al., 2017; Tishkoff et al., 2009). Identifying genetic variants associated with BP enhances our understanding of BP regulation and might highlight potential drug targets for hypertension treatment and prevention. Furthermore, the identification of variants associated with hypertension in both adolescence and adulthood could offer opportunities for early risk prediction.

## 2 METHODS

### 2.1 Ethical compliance

This study was approved by the Uganda Virus Research Institute Science and Ethics Committee; the Uganda National Council for Science & Technology; the London School of Hygiene & Tropical Medicine; and the Oxford Tropical Research Ethics Committee. Written informed assent and consent were obtained.

### 2.2 Study design, population and setting

The EMaBS [trial registration ISRCTN32849447] in Uganda, was originally designed to investigate the influence of worms and their treatment in pregnancy and early childhood on vaccine response and on infections in childhood (Elliott et al., 2007). Briefly, between April 2003 and November 2005, 2,507 pregnant women in their second or third trimester were randomized in a 2 × 2 factorial design to receive single dose albendazole (400 mg) or matching placebo and single dose praziquantel (40 mg/kg) or matching placebo. At 15 months of age, the resulting 2,345 live‐born infants were randomized to receive quarterly albendazole or matching placebo up to 5 years of age.

### 2.3 Phenotyping

The offspring continued under follow‐up after the trial ended in 2011. From 20 May 2014 to 16 June 2016, cohort participants who were now aged 10–11 years were enrolled in a BP sub‐study, as part of which additional anthropometric and BP data were collected (Lule, Namara, Akurut, Muhangi, et al., 2019). Adolescents were included in this study if they were aged 10 or 11 years and attending their routine annual follow‐up visit during the BP sub‐study period (11‐year‐olds who had previously enrolled as 10‐year‐olds were not included twice). Where necessary, enrollment was postponed until the participant was free of malaria (fever or axillary temperature ≥37.5°C and parasitemia) and other illnesses (Lule, Namara, Akurut, Muhangi, et al., 2019).

BP was measured as previously described (Lule, Namara, Akurut, Muhangi, et al., 2019). Briefly, on the BP study visit day, after 5 min rest period, trained nurses measured BP thrice 5 min apart, on the right arm supported at the heart level, with the participant seated upright all the way to the back of the chair, with legs uncrossed and feet flat on the floor. Automated Omron (M6, HEM‐700) machines validated every 6 months by the Uganda National Bureau of Standards were used. Blood pressure phenotypes for this analysis were the mean of the second and third readings for systolic and diastolic BP, that is, systolic and diastolic BP were analyzed as two separate phenotypes. The second and third BP readings were, on average, lower than the first BP reading but similar to each other for both systolic and diastolic BP (Lule, Namara, Akurut, Muhangi, et al., 2019).

### 2.4 Genotyping and quality control

Earlier in 2013, whole‐genome genotyping of 1,391 EMaBS participants was undertaken. Genotypic data were generated from red cell pellets that had been separated and stored at −80°C until processing. Approximately 2.2 million genetic variants were generated at Wellcome Sanger Institute using the Illumina HumanOmni2.5M‐8 (“octo”) Beadchip arrays, version 1.1 (Illumina Inc.). Quality control (using standard pipelines) was performed at the University of Oxford using commands in PLINK (version 1.7; Purcell et al., 2007) to remove individuals and variants with high levels of missingness or deviations from expected levels of heterozygosity or Hardy–Weinberg equilibrium (*p* < 1 × 10^−8^). Untyped genetic variants and the variants identified for replication analysis were imputed in the EMaBS sample using a merged panel (1,000 Genomes Project Consortium et al., 2015, African genome variation project [AGVP] Gurdasani et al., 2015 and Uganda 2000 Genomes [UG2G]: genomes of Ugandan individuals of diverse ethnicity from rural Uganda) at the Wellcome Centre for Human Genetics. SHAPEIT2 (version 2 790; O'Connell et al., 2014), and IMPUTE2 (version 2.3.2; Marchini, Howie, Myers, McVean, & Donnelly, 2007) were used for imputation using settings as recommended for African populations. Only SNPs with an INFO score >0.3 and a minor allele frequency >0.01 were taken forward for analysis.

### 2.5 Association analysis

The analysis included EMaBS adolescents with phenotypic and genotypic data. The two outcomes (mean systolic BP and mean diastolic BP) were analyzed separately. GWAS of BP (systolic and diastolic) as quantitative traits was done using mixed linear regression methods (accounting for population substructure) assuming an additive model and controlling for age and body mass index (BMI) as covariates in genome‐wide complex trait analysis (GCTA) version 1.22 (Yang, Lee, Goddard, & Visscher, 2011). A *p* < 5 × 10^−8^ was considered as the threshold to denote genome‐wide significance for SNPs. Results for SNPs with *p* < 1 × 10^−6^ are reported. Manhattan plots and Quantile–Quantile (Q–Q) plots were constructed to show the distributions of association *p*‐values and the departure of the observed *p*‐values from the null, respectively.

For the replication component of this study, previously published BP GWASs were searched to identify SNPs reported to be associated with systolic or diastolic BP (*p* < 5 × 10^−8^ in the original GWAS) and these variants were considered for the replication. Replication analysis was conducted using linear regression adjusting for age and BMI in Stata version 14 (College Station, Texas, USA). Variants were tested for association with the phenotype they were associated with in the published GWAS, that is variants associated with systolic BP in a published GWAS were tested for association with systolic BP but not with diastolic BP and vice versa. *P* < .05 was considered the threshold for statistical significance for the replication study although results were also interpreted in light of a Bonferroni correction allowing for all tests done in the replication analysis.

The base pair position is based on the Genome Reference Consortium Human Build 37, February 2009 (GRCh37/hg19).

## 3 RESULTS

The discovery GWAS analysis used data on 20,074,711 SNPs from 815 adolescents. These adolescents had a mean age of 10.4 years, a mean BMI of 16.0 kg/m^2^, mean systolic BP of 106.0 mmHg, and mean diastolic BP of 65.3 mmHg. Four hundred and seventeen (51%) of the adolescents were male. Detailed characteristics of EMaBS participants included and not included in the analysis are described in Table S1. Offspring included in the genetic analysis were similar for most characteristics to those not included, except that those included were more likely to have been delivered in Entebbe hospital than elsewhere and to have been exclusively breastfeeding at 6 weeks of age.

**Figure 1:**
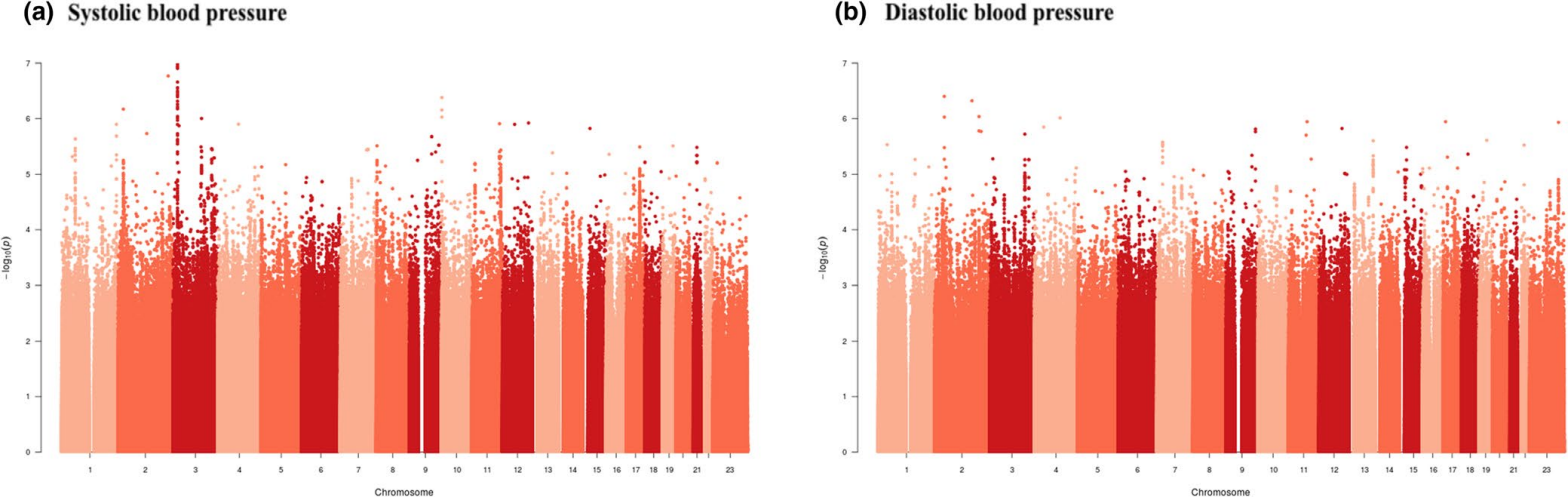
Manhattan plots for the association of SNPs with (a) systolic blood pressure and (b) diastolic blood pressure adjusting for age and body mass index as fixed covariates

**Figure 2:**
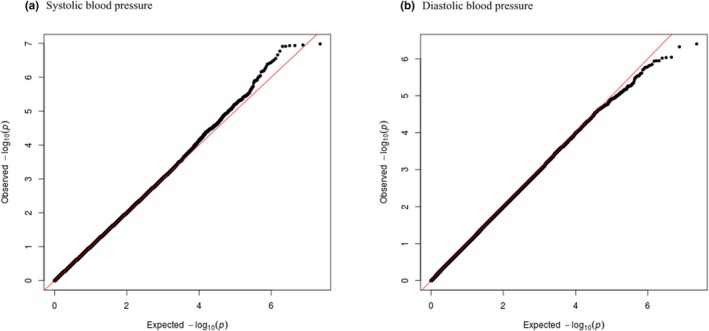
Quantile–Quantile (Q–Q) plots for the two phenotypes and the genomic control coefficient (lambda). (a) systolic blood pressure and (b) diastolic blood pressure

The distributions of association *p*‐values (Manhattan plot) for systolic and diastolic BP phenotypes are shown in Figure 1 and the Q–Q plots in Figure 2. The observed P‐values show no departure from the null (Figure 2), either for systolic or diastolic BP, with lambda values of 1.006 and 0.995, respectively. The results show adequate control for population substructure in the analysis.

The SNPs most strongly associated with BP are shown in Table 1. None of the SNPs reached genome‐wide level of significance (*p* < 5 × 10^−8^) for association with adolescent systolic or diastolic BP. Borderline significance (5 × 10^−8^ < *p* < 1 × 10^−6^) for association with systolic BP was achieved for four index SNPs at four separate loci; there were four SNPs showing borderline significant associations with diastolic BP at four separate loci. There was no overlap between the SNPs most strongly associated with systolic BP and those most strongly associated with diastolic BP. None of these index SNPs have been identified as associated with BP in previously published BP GWAS. The most strongly associated SNP for systolic BP with *p*‐value 6.8 × 10^−7^ was rs181430167, located in the intron region of *KLHL29* on chromosome 2. The lowest *p*‐value (4.0 × 10^−7^) for association with diastolic BP was for rs12991132 located between *ZFP36L2, THADA, LOC1001297261* on chromosome 2.

**Table 1:** Genome‐wide association study results: SNPs associated with blood pressure (systolic or diastolic) at *p* < 1.0 × 10^−7^

Chr	Nearest gene^a^	Position^b^	SNP	Distance to gene (kb)	Type	EA	RA	EAF	β	*SE*	*p*‐value
Systolic blood pressure											
2	KLHL29	23904233	rs181430167	0	Intron	C	T	0.05	4.62	9.24	6.8 × 10^−7^
10	LINC00701	2565732	rs71502208	500	Unknown	A	G	0.22	2.51	5.05	9.3 × 10^−7^
3	SGOL1/SGOL‐ASI	20295100	rs139992073	200	Unknown	C	T	0.14	2.83	5.74	9.7 × 10^−7^
3	PLXNA1	126737466	rs73861745	0	Intron	A	G	0.26	2.25	4.57	9.9 × 10^−7^
Diastolic blood pressure											
2	ZFP36L2/THADA/LOC1001297261	43291689	rs12991132	500	Unknown	A	G	0.59	−1.87	3.61	4.0 × 10^−7^
2	COBLL	165534347	rs111770209	20	Unknown	T	C	0.94	−3.82	7.53	4.8 × 10^−7^
2	DNAH7	196853830	rs13403027	0	Intron	A	G	0.71	−1.91	3.87	9.1 × 10^−7^
4	ANK2	114209732	rs29356	0	Intron	T	C	0.69	1.91	3.87	9.6 × 10^−7^

Abbreviations: Chr, chromosomes; EA, effect allele; EAF, effect allele frequency; RA, reference allele; SE, standard error; SNP, single nucleotide polymorphism; β, effect size estimates correspond to mean difference in mmHg per effect allele for systolic or diastolic blood pressure, adjusted for age and body mass index.

a Named according to the nearest annotated gene(s).

b Given with respect to Build 37 (GRCh37/hg19).

Of the 389 SNPs (Adeyemo et al., 2009; Cho et al., 2009; Ehret et al., 2016; International Consortium for Blood Pressure Genome‐Wide Association Studies et al., 2011; Evangelou et al., 2018; Fox et al., 2011; Franceschini et al., 2013; Ganesh et al., 2014, 2013; Ho et al., 2011; Hoffmann et al., 2017; Johnson, Newton‐Cheh, et al., 2011; Johnson, Gaunt, et al., 2011; Kato et al., 2015, 2011; Kidambi et al., 2012; Levy et al., 2009; Li et al., 2017; Liang et al., 2017; Liu et al., 2016; Newton‐Cheh, Johnson, et al., 2009; Newton‐Cheh, Larson, et al., 2009; Parmar et al., 2016; Simino et al., 2014; Surendran et al., 2016; Takeuchi et al., 2010; Tragante et al., 2014; Wain et al., 2017, 2011; Warren et al., 2017) identified for replication, 330 (85%) SNPs were included in the replication analysis. Fifty‐nine SNPs that were either rare (<0.01) or poorly imputed (INFO score <0.3) in the EMaBS sample were not included in the replication analysis. Thirty SNPs of the 330 SNPs included in the replication had been previously associated with BP in populations of African origins. Forty SNPs had been previously associated with both systolic and diastolic BP and were tested for association with both. Tables 2 and 3 show results from the replication analysis. Briefly, 33 SNPs (17 for systolic, 15 for diastolic and one for both systolic and diastolic BP) were associated with BP in this population, with the same effect direction as the discovery population for 14 of the SNPs (five for systolic BP and eight for diastolic BP and one for both systolic and diastolic BP).

**Table 2:** Loci associated with systolic blood pressure identified from previous GWAS and results of replication in Entebbe Mother and Baby Study (EMaBS) sample

Chr	SNP	Position^a^	Nearest gene	Population	EA/RA	Discovery sample				EMaBS sample			
						EAF	β	*SE*	*p*‐value	EAF	β^b^	*SE*	*p*‐value
1	rs839755	43856410	SZT2	E	A/C	0.62	−0.27	0.03	5.4 × 10^‒18^	0.81	0.29	0.70	6.8 × 10^‒1^
**1**	**rs4926499**	**249155909**	**AL672294.1**	**E**	**C/G**	**0.82**	**0.30**	**0.04**	**1.3 × 10^‒11^**	**0.98**	**−5.12**	**2.00**	**1.1 × 10^‒2^**
1	rs1043069	180859368	XPR1	E	T/G	0.62	0.23	0.03	5.2 × 10^‒14^	0.63	0.02	0.62	9.7 × 10^‒1^
1	rs4651224	184585182	C1orf21	E	T/C	0.45	0.20	0.03	9.0 × 10^‒11^	0.94	0.75	1.24	5.5 × 10^‒1^
1	rs2807337	22577371	WNT4	E	T/C	0.37	0.19	0.03	2.8 × 10^‒9^	0.43	−0.70	0.58	2.3 × 10^‒1^
**1**	**rs7514579**	**94051350**	**BCAR3**	**E**	**A/C**	**0.77**	**0.22**	**0.03**	**5.5 × 10^‒10^**	**0.50**	**1.25** c	**0.67**	**4.0 × 10^‒2^**
1	rs17396055	94730954	ARHGAP29	E	A/G	0.33	−0.17	0.03	4.0 × 10^‒8^	0.08	0.80	1.02	4.3 × 10^‒1^
1	rs12042924	197297417	CRB1	E	T/C	0.53	−0.18	0.03	2.6 × 10^‒9^	0.86	−1.29	0.86	1.3 × 10^‒1^
1	rs7555285	209970355	IRF6	E	C/G	0.80	0.23	0.04	1.1 × 10^‒9^	0.83	−0.52	0.77	5.0 × 10^‒1^
1	rs33996239	203109801	ADORA1	E	T/C	0.06	−0.37	0.07	3.4 × 10^‒8^	0.09	−0.13	1.08	9.0 × 10^‒1^
1	rs2932538	113216543	MOV10	E	G/A	0.75	0.39	—	1.2 × 10^‒9^	0.85	0.15	0.80	8.6 × 10^‒1^
1	rs7515635	42408070	HIVEP3	E	T/C	0.47	0.31	0.04	4.8 × 10^‒12^	0.68	0.08	0.65	9.0 × 10^‒1^
1	rs17367504	11862778	MTHFR‐NPPB	E	G/A	0.14	−0.85	0.11	2.0 × 10^‒13^	0.09	0.66	1.10	5.5 × 10^‒1^
1	rs2493292	3328659	PRDM16	E/AA	T/C	0.15	0.37	0.07	1.4 × 10^‒8^	0.17	−0.36	0.82	6.6 × 10^‒1^
1	rs880315	10796866	CASZ1	AS	C/T	0.34	1.08	0.03	2.2 × 10^‒8^	0.11	−0.91	0.96	3.5 × 10^‒1^
1	rs3820068	15798197	CELA2A	E	A/G	0.81	0.43	0.06	1.1 × 10^‒8^	0.63	0.81	0.63	2.0 × 10^‒1^
1	rs10922502	89360158	GTF2B	E	A/G	0.62	−0.38	0.05	2.2 × 10^‒15^	0.67	−0.32	0.64	6.1 × 10^‒1^
**2**	**rs2972146**	**227100698**	**2q36.3**	**E**	**T/G**	**0.65**	**0.17**	—	**8.4 × 10^‒9^**	**0.86**	**−0.19**	**0.86**	**4.0 × 10^‒2^**
2	rs1446468	164963486	FIGN‐GRB14	E	T/C	0.53	−0.50	0.07	1.8 × 10^‒12^	0.95	0.46	1.34	7.3 × 10^‒1^
2	rs16849225	164906820	FIGN‐GRB14	AS	C/T	0.61	0.75	0.11	3.5 × 10^‒11^	0.89	−1.04	0.93	2.7 × 10^‒1^
2	rs7562	28635740	FOSL2	E	T/C	0.52	0.26	0.05	1.9 × 10^‒8^	0.26	0.20	0.67	7.6 × 10^‒1^
2	rs13420463	37517566	PRKD3	E	A/G	0.77	0.36	0.05	7.0 × 10^‒11^	0.45	0.03	0.63	9.6 × 10^‒1^
2	rs55780018	208526140	METTL21A‐AC079767.3	E	T/C	0.54	−0.39	0.05	5.9 × 10^‒16^	0.75	0.59	0.74	4.2 × 10^‒1^
**2**	**rs1275988**	**26914364**	**KCNK3**	**E**	**T/C**	**0.23**	**−0.60**	**0.09**	**2.6 × 10^‒10^**	**0.14**	**2.01**	**0.85**	**1.8 × 10^‒2^**
**2**	**rs6712094**	**165043460**	**FIGN‐GRB14**	**E**	**A/G**	**0.70**	**0.60**	**0.10**	**9.9 × 10^‒9^**	**0.89**	**−3.72**	**1.01**	**2.6 × 10^‒4^**
2	rs1344653	19730845	OSR1	E/AS	A/G	0.54	−0.27	0.04	7.8 × 10^‒12^	0.67	0.07	0.66	9.1 × 10^‒1^
2	rs2300481	66782467	MEIS1	E	T/C	0.39	0.20	0.03	1.6 × 10^‒10^	0.37	0.73	0.61	2.3 × 10^‒1^
2	rs35590893	43716933	HADA	E	A/G	0.27	−0.24	0.03	1.7 × 10^‒12^	0.13	0.52	0.86	5.5 × 10^‒1^
2	rs67720684	18975439	NT5C1B	E	A/C	0.24	0.19	0.04	3.8 × 10^‒8^	0.36	−0.41	0.60	5.0 × 10^‒1^
2	rs28377357	112769721	MERTK	E	A/G	0.29	−0.21	0.03	9.6 × 10^‒11^	0.31	0.87	0.64	1.8 × 10^‒1^
2	rs72816333	60096560	RP11−444A22.1	E	A/T	0.83	0.23	0.04	5.5 × 10^‒9^	0.96	−0.15	1.48	9.2 × 10^‒1^
2	rs28558491	187816321	ZSWIM2	E	T/C	0.74	−0.21	0.03	7.5 × 10^‒10^	0.29	0.55	0.67	4.1 × 10^‒1^
2	rs6723509	122000745	TFCP2L1	E	T/C	0.86	0.25	0.04	7.6 × 10^‒9^	0.91	0.78	1.00	4.4 × 10^‒1^
2	rs1044822	230629138	TRIP12	E	T/C	0.15	−0.25	0.04	5.2 × 10^‒9^	0.09	−0.41	1.07	7.0 × 10^‒1^
2	rs12694277	213188795	ERBB4	E	T/C	0.30	−0.20	0.03	1.8 × 10^‒9^	0.62	−0.05	0.61	9.4 × 10^‒1^
2	rs6739913	185033065	ZNF804A	E	A/G	0.28	0.18	0.03	6.5 × 10^‒8^	0.21	0.21	0.71	7.6 × 10^‒1^
**2**	**rs2920899**	**55279681**	**RTN4**	**E**	**T/G**	**0.79**	**0.20**	**0.04**	**9.5 × 10^‒8^**	**0.86**	**1.58** c	**0.78**	**4.0 × 10^‒2^**
3	rs9810888	53635595	CACNA1D	AS	G/T	0.39	0.53	0.10	5.5 × 10^‒8^	0.60	−1.15	0.60	5.4 × 10^‒2^
3	rs11128722	14958126	FGD5	E	A/G	0.56	−0.31	0.05	3.6 × 10^‒11^	0.32	0.39	0.10	5.2 × 10^‒1^
3	rs9859176	134000025	RYK	E	T/C	0.40	0.32	0.05	1.3 × 10^‒11^	0.17	0.57	0.77	4.6 × 10^‒1^
3	rs419076	169100886	MECOM	E	T/C	0.47	0.41	—	1.8 × 10^‒13^	0.57	−0.60	0.61	3.2 × 10^‒1^
3	rs347591	11290122	HRH1	E/AS/AA	G/T	0.35	−0.53	0.11	1.5 × 10^‒8^	0.57	−0.41	0.60	4.9 × 10^‒1^
3	rs13082711	27537909	SL4A7	E	T/C	0.78	−0.24	—	3.8 × 10^‒9^	0.95	−0.33	1.41	8.1 × 10^‒1^
3	rs319690	47927484	MAP4	E	T/C	0.50	0.42	0.07	4.7 × 10^‒8^	0.44	0.72	0.62	2.5 × 10^‒1^
3	rs12638085	30405936	TGFBR2	E	A/T	0.35	0.22	0.03	5.6 × 10^‒12^	0.12	−1.31	0.92	1.6 × 10^‒1^
3	rs6788984	41107173	CTNNB1	E	A/G	0.86	0.30	0.04	3.8 × 10^‒12^	0.71	−0.05	0.66	9.4 × 10^‒1^
**3**	**rs9875380**	**132780356**	**TMEM108**	**E**	**T/C**	**0.46**	**−0.18**	**0.03**	**6.5 × 10^‒9^**	**0.26**	**−1.55** c	**0.68**	**2.3 × 10^‒2^**
3	rs863930	135949737	PCCB	E	A/C	0.54	0.19	0.03	5.1 × 10^‒10^	0.63	−0.77	0.62	2.2 × 10^‒1^
3	rs78151625	158316726	MLF1	E	T/C	0.83	−0.25	0.04	1.6 × 10^‒9^	−0.82	3.49	3.49	8.1 × 10^‒1^
3	rs6774721	49381898	ARIH2	E	C/T	0.88	0.28	0.05	6.4 × 10^‒9^	0.83	−0.94	0.85	3.7 × 10^‒1^
3	rs9857362	74710462	CNTN3	E	A/C	0.53	0.17	0.03	1.6 × 10^‒8^	0.83	0.65	0.77	3.9 × 10^‒1^
4	rs1458038	81164723	FGF5	E	T/C	0.29	0.71	—	1.5 × 10^‒23^	0.04	1.28	1.53	4.1 × 10^‒1^
4	rs2291435	38387395	TBC1D1‐FLJ13197	E/AA	T/C	0.52	−0.34	0.04	1.9 × 10^‒14^	0.30	−0.54	0.64	4.0 × 10^‒1^
4	rs13112725	106911742	NPNT	E	C/G	0.76	0.44	0.06	1.5 × 10^‒14^	0.61	−0.62	0.60	3.0 × 10^‒1^
4	rs231708	2694773	FAM193A	E	C/G	0.69	−0.12	0.03	4.7 × 10^‒18^	0.24	1.14	0.68	9.6 × 10^‒2^
4	rs7439567	138464842	P11−714L20.1	E	T/C	0.42	0.25	0.03	2.3 × 10^‒16^	0.81	−0.58	0.76	4.4 × 10^‒1^
4	rs2610990	18008232	LCORL	E	A/G	0.26	−0.29	0.03	2.8 × 10^‒17^	0.19	−1.19	0.77	1.2 × 10^‒1^
**4**	**rs17035181**	**157678511**	**PDGFC**	**E**	**T/G**	**0.85**	**0.31**	**0.04**	**7.6 × 10^‒13^**	**0.75**	**−1.47**	**0.71**	**3.8 × 10^‒2^**
4	rs1347345	95938386	MPR1B	E	A/G	0.62	−0.18	0.03	6.9 × 10^‒9^	0.87	0.23	0.88	8.0 × 10^‒1^
4	rs12511987	46595623	GABRA2	E	T/G	0.82	−0.23	0.04	5.4 × 10^‒9^	0.94	−0.53	1.45	7.2 × 10^‒1^
4	rs2014912	86715670	ARHGAP24	E/AS	T/C	0.16	0.62	0.08	5.4 × 10^‒17^	0.16	1.16	0.82	1.6 × 10^‒1^
5	rs13359291	122476457	PRDM6	E/AS	A/G	0.31	0.53	0.07	8.9 × 10^‒16^	0.16	0.66	0.77	3.9 × 10^‒1^
5	rs1173771	32815028	NPR3‐C5orf23	E	G/A	0.60	0.50	—	1.8 × 10^‒16^	0.82	1.09	0.82	1.9 × 10^‒1^
5	rs11953630	157845402	EBF1	E	T/C	0.37	−0.41	—	3.0 × 10^‒11^	0.13	−0.34	0.88	7.0 × 10^‒1^
5	rs10077885	114390121	TRIM36	E	A/C	0.50	−0.28	0.04	1.6 × 10^‒10^	0.65	0.20	0.63	7.5 × 10^‒1^
5	rs6595838	127868199	FBN2	E	A/G	0.30	0.34	0.05	7.6 × 10^‒12^	0.63	0.62	0.59	2.9 × 10^‒1^
5	rs1173766	32804528	NPR3	AS	C/T	0.60	0.63	0.11	1.9 × 10^‒8^	0.66	1.13	0.64	7.8 × 10^‒1^
5	rs10069690	1279790	TERT	E	T/C	0.26	0.31	0.04	4.8 × 10^‒17^	0.66	−0.54	0.65	4.0 × 10^‒1^
5	rs709668	96174186	CTD−2260A17.2	E	A/G	0.20	−0.29	0.04	6.0 × 10^‒15^	0.40	−0.43	0.59	4.7 × 10^‒1^
5	rs246973	68007803	SLC30A5	E	T/C	0.29	0.25	0.03	1.5 × 10^‒13^	0.40	2.51	1.53	1.0 × 10^‒1^
5	rs702395	140086677	ZMAT2	E	T/C	0.44	0.23	0.03	3.5 × 10^‒14^	0.29	−0.15	0.65	8.2 × 10^‒1^
5	rs13179413	55868097	AC022431.2	E	T/C	0.28	0.22	0.03	1.1 × 10^‒10^	0.21	−0.23	0.72	7.5 × 10^‒1^
**5**	**rs62373688**	**127352807**	**CTC−228N24.3**	**E**	**A/T**	**0.13**	**0.27**	**0.04**	**1.5 × 10^‒9^**	**0.24**	**1.40** c	**0.70**	**4.6 × 10^‒2^**
5	rs74774746	33411769	TARS	E	C/G	0.26	−0.19	0.04	5.6 × 10^‒8^	0.14	1.49	0.88	9.2 × 10^‒2^
5	rs1008058	122435627	PRDM6	E	A/G	0.14	0.55	—	3.0 × 10^‒10^	0.13	0.31	0.89	7.3 × 10^‒1^
6	rs79030490	134087689	TARID‐TCF21	AA	A/C	0.09	−1.83	0.31	3.0 × 10^‒9^	0.11	0.99	1.19	4.0 × 10^‒1^
6	rs76987554	134080855	TARID‐TCF21	AA	C/T	0.91	1.85	0.31	2.2 × 10^‒9^	0.90	−0.88	1.20	4.6 × 10^‒1^
6	rs1799945	26091179	HFE	E	G/C	0.14	0.63	—	7.7 × 10^‒12^	0.02	−1.84	2.17	4.0 × 10^‒1^
**6**	**rs805303**	**31616366**	**BAT2‐BAT5**	**E**	**G/A**	**0.61**	**0.38**	—	**1.5 × 10^‒11^**	**0.40**	**1.66** c	**0.61**	**7.0 × 10^‒3^**
6	rs6911827	22130601	CASC15	E	T/C	0.45	0.30	0.05	2.0 × 10^‒10^	0.82	0.81	0.81	3.2 × 10^‒1^
6	rs2270860	43270151	SLC22A7	E/AA	T/C	0.37	0.32	0.05	2.9 × 10^‒11^	0.78	−1.20	0.71	9.1 × 10^‒2^
**6**	**rs1563788**	**43308363**	**TTBK1‐SLC22A7‐ZNF318**	**E/AS**	**T/C**	**0.31**	**0.51**	**0.06**	**2.2 × 10^‒16^**	**0.78**	**−1.46**	**0.71**	**4.0 × 10^‒2^**
6	rs13209747	127115454	RSPO3	E/AA/AS	T/C	0.19	0.85	0.21	2.6 × 10^‒10^	0.07	−0.13	1.09	9.1 × 10^‒1^
6	rs17080102	151004770	PLEKHG1	E/AA/AS	C/G	0.10	−1.02	0.25	4.8 × 10^‒8^	0.15	−0.61	0.81	4.5 × 10^‒1^
6	rs9368222	20686996	CDKAL1	E	A/C	0.27	0.23	0.03	1.8 × 10^‒11^	0.17	−0.07	0.81	9.3 × 10^‒1^
6	rs10782230	126228512	NCOA7	E	A/G	0.48	0.21	0.03	2.9 × 10^‒12^	0.41	0.71	0.62	2.6 × 10^‒1^
6	rs2745599	1613686	FOXC1	E	A/G	0.55	0.22	0.03	9.8 × 10^‒12^	0.10	−1.48	0.95	1.2 × 10^‒1^
6	rs9885632	131311909	EPB41L2	E	T/C	0.73	0.24	0.03	4.3 × 10^‒12^	0.94	0.64	1.39	6.4 × 10^‒1^
6	rs7763294	140383733	CITED2	E	T/G	0.32	−0.20	0.03	6.4 × 10^‒10^	0.10	0.32	0.97	7.4 × 10^‒1^
7	rs2969070	2512545	CHST12‐LFNG	E	A/G	0.63	−0.30	0.05	1.4 × 10^‒10^	0.97	−0.01	1.80	1.0 × 10^‒0^
7	rs11556924	129663496	ZC3HC1	E	T/C	0.38	−0.28	0.05	7.6 × 10^‒9^	0.01	−0.24	1.58	8.8 × 10^‒1^
7	rs13238550	131059056	MKLN1	E	A/G	0.40	0.33	0.05	1.9 × 10^‒12^	0.09	−0.14	1.14	9.0 × 10^‒1^
7	rs1011018	139463264	HIPK2	E	A/G	0.20	−0.33	0.06	1.5 × 10^‒8^	0.61	−0.17	0.59	7.8 × 10^‒1^
7	rs4728142	128573967	7q32.1	E	A/G	0.43	−0.24	‐	3.5 × 10^‒8^	0.23	−0.27	0.69	7.0 × 10^‒1^
7	rs17477177	106411858	PIK3CG	E	T/C	0.72	−0.55	0.08	5.7 × 10^‒11^	0.94	−0.86	1.15	4.5 × 10^‒1^
7	rs17428471	27337867	EVX1‐HOXA	E/AA/AS	T/G	0.14	1.20	0.24	2.1 × 10^‒12^	0.13	1.39	0.90	1.3 × 10^‒1^
7	rs11563582	27351650	EVX1‐HOXA	AA	A/G	0.13	1.61	0.28	7.1 × 10^‒9^	0.17	0.56	0.81	4.9 × 10^‒1^
7	rs848445	77572461	PHTF2	E	T/C	0.23	−0.20	0.03	2.3 × 10^‒9^	0.08	−0.09	1.03	9.3 × 10^‒1^
7	rs6963105	75097488	POM121C	E	A/G	0.43	−0.19	0.03	3.8 × 10^‒9^	0.06	0.17	1.15	8.8 × 10^‒1^
7	rs10274928	28142088	JAZF1	E	A/G	0.49	0.16	0.03	8.2 × 10^‒8^	0.66	−0.05	0.63	9.3 × 10^‒1^
7	rs11771693	150050111	RARRES2	E	A/G	0.67	0.18	0.03	1.9 × 10^‒8^	0.52	−0.25	0.62	6.8 × 10^‒1^
8	rs4841569	11452177	BLK‐GATA4	E/AS	G/A	0.51	0.47	0.02	5.6 × 10^‒10^	0.91	0.33	1.13	7.7 × 10^‒1^
8	rs2898290	11433909	BLK‐GATA4	E	T/C	0.53	0.53	0.80	3.2 × 10^‒8^	0.61	−0.71	0.60	2.3 × 10^‒1^
**8**	**rs1986971**	**10268736**	**MSRA**	**E**	**A/G**	**0.70**	**0.26**	**0.03**	**1.6 × 10^‒14^**	**0.80**	**1.45** c	**0.73**	**4.8 × 10^‒2^**
8	rs1906672	38130025	WHSC1L1	E	A/G	0.23	0.30	0.04	1.2 × 10^‒16^	0.16	0.08	0.82	9.2 × 10^‒1^
8	rs72688070	81393697	Y_RNA	E	T/C	0.17	−0.27	0.04	2.8 × 10^‒11^	0.44	−0.62	0.60	3.0 × 10^‒1^
**8**	**rs62491354**	**9730663**	**TNKS**	**E**	**A/G**	**0.13**	**0.31**	**0.04**	**3.3 × 10^‒12^**	**0.13**	**−2.38**	**0.80**	**3.0 × 10^‒3^**
8	rs4129585	143312933	TSNARE1	E	A/C	0.44	0.19	0.03	1.0 × 10^‒9^	0.06	−1.10	1.18	3.5 × 10^‒1^
8	rs6557876	25900675	EBF2	E	C/T	0.25	−0.37	0.05	2.8 × 10^‒14^	0.50	−0.20	0.60	7.4 × 10^‒1^
8	rs894344	135612745	ZFAT	E	A/G	0.60	−0.26	0.05	3.2 × 10^‒8^	0.65	−0.17	0.64	8.0 × 10^‒1^
9	rs10760117	123586737	PSMD5	E	T/G	0.42	0.28	0.05	6.1 × 10^‒10^	0.78	−1.39	0.72	5.3 × 10^‒2^
9	rs1332813	9350706	PTPRD	E	T/C	0.35	0.22	0.03	2.3 × 10^‒12^	0.36	0.02	0.62	9.7 × 10^‒1^
**9**	**rs7045409**	**95201540**	**CENPP**	**E**	**A/T**	**0.37**	**−0.19**	**0.03**	**2.6 × 10^‒9^**	**0.90**	**2.16**	**0.94**	**2.2 × 10^‒2^**
9	rs1891730	130309028	FAM129B	E	T/C	0.62	−0.18	0.03	7.7 × 10^‒9^	0.39	−1.13	0.61	6.2 × 10^‒2^
9	rs28558845	4334791	GLIS3	E	C/G	0.16	−0.26	0.04	1.2 × 10^‒9^	0.24	0.29	0.72	6.9 × 10^‒1^
10	rs1133400	134459388	INPP5A	E	A/G	0.79	−0.30	0.04	2.5 × 10^‒15^	0.86	0.11	0.88	9.0 × 10^‒1^
10	rs11191548	104846178	CYP17A1‐NT5C2	E	T/C	0.91	1.16	0.12	7.0 × 10^‒24^	0.98	−0.41	1.62	8.0 × 10^‒1^
**10**	**rs112184198**	**102604514**	**PAX2**	**E**	**A/G**	**0.10**	**−0.66**	**0.08**	**3.6 × 10^‒18^**	**0.06**	**3.67**	**1.37**	**8.0 × 10^‒3^**
10	rs1813353	18707448	CACNB2	E	T/C	0.68	0.57	—	2.6 × 10^‒12^	0.84	−0.74	0.84	3.8 × 10^‒1^
10	rs932764	95895940	PLCE1	E	G/A	0.44	0.48	—	7.1 × 10^‒16^	0.15	0.59	0.85	4.9 × 10^‒1^
10	rs1801253	115805056	ADRB1	E	G/C	0.27	−0.57	0.09	4.7 × 10^‒10^	0.34	0.21	0.64	7.5 × 10^‒1^
10	rs4387287	105677897	OBFC1	E/AS	A/C	0.16	0.36	—	9.1 × 10^‒10^	0.73	−0.00	0.67	1.0 × 10^‒0^
10	rs4590817	63467553	C10orf107	E	G/C	0.84	0.65	—	4.0 × 10^‒12^	0.84	−0.55	0.78	4.8 × 10^‒1^
10	rs7912283	133773019	PPP2R2D	E	A/G	0.35	0.21	0.03	6.4 × 10^‒11^	0.89	−0.31	0.89	7.3 × 10^‒1^
10	rs12572586	74751579	PLA2G12B	E	T/C	0.94	−0.39	0.06	1.2 × 10^‒9^	0.95	1.91	1.41	1.8 × 10^‒1^
10	rs11197813	118523933	HSPA12A	E	A/G	0.70	−0.18	0.03	3.5 × 10^‒8^	0.82	0.97	0.74	1.9 × 10^‒1^
10	rs4373814	18419972	CACNB2	E	G/C	0.55	−0.37		4.8 × 10^‒11^	0.39	−0.82	0.60	1.7 × 10^‒1^
11	rs7103648	47461783	RAPSN‐PSMC3‐SLC39A13	E	A/G	0.61	−0.33	0.05	4.4 × 10^‒13^	0.85	0.97	0.85	2.6 × 10^‒1^
11	rs751984	61278246	LRRC10B	E	T/C	0.88	0.41	0.07	3.8 × 10^‒9^	0.79	0.04	0.72	9.5 × 10^‒1^
11	rs661348	1905292	LSP1‐TNNT3	E	T/C	0.57	−0.65	0.11	7.0 × 10^‒10^	0.86	0.89	0.98	3.3 × 10^‒1^
11	rs7129220	10350538	ADM	E	G/A	0.89	−0.62	—	3.0 × 10^‒12^	0.92	−1.02	1.16	3.8 × 10^‒1^
11	rs633185	100593538	FLJ32810‐TMEM133	E	G/C	0.28	−0.57	—	1.2 × 10^‒17^	0.23	−0.92	0.71	2.0 × 10^‒1^
11	rs4757391	16302939	SOX6	E/AS/AA	T/C	0.21	0.56	0.12	5.7 × 10^‒10^	0.74	0.19	0.65	7.7 × 10^‒1^
11	rs11229457	58207203	OR5B12	E/AS	T/C	0.24	−0.31	—	2.7 × 10^‒8^	0.29	−0.36	0.62	5.6 × 10^‒1^
11	rs381815	16902268	PLEKHA7	E	T/C	0.26	0.57	—	5.3 × 10^‒11^	0.26	−0.27	0.71	5.6 × 10^‒1^
11	rs3741378	65408937	RELA	E	T/C	0.14	−0.55	—	3.4 × 10^‒10^	0.76	−1.04	0.69	1.3 × 10^‒1^
11	rs4385883	51539339	TRIM48	E	T/A	0.29	−0.25	0.04	1.4 × 10^‒12^	0.53	−0.10	0.60	8.6 × 10^‒1^
11	rs11041530	7701503	CYB5R2	AA	C/G	0.11	−1.35	0.25	4.0 × 10^‒8^	0.17	0.49	0.82	5.5 × 10^‒1^
11	rs1401454	16250183	SOX6	AA	T/C	0.46	0.55	0.16	5.6 × 10^‒8^	0.46	−0.60	0.59	3.1 × 10^‒1^
11	rs7941684	5532222	UBQLN3	AA	T/G	0.80	−1.23	0.22	2.4 × 10^‒8^	0.82	−0.69	0.80	3.8 × 10^‒1^
11	rs11031051	30355707	ARL14EP	E	A/C	0.69	−0.22	0.03	7.7 × 10^‒12^	0.58	0.26	0.61	6.7 × 10^‒1^
**11**	**rs67976715**	**68023742**	**C11orf24**	**E**	**C/G**	**0.23**	**0.21**	**0.04**	**6.8 × 10^‒9^**	**0.07**	**−2.44**	**1.23**	**4.8 × 10^‒2^**
11	rs10743086	8774923	ST5	E	A/G	0.21	−0.21	0.04	3.6 × 10^‒8^	0.33	0.96	0.61	1.2 × 10^‒1^
12	rs11067763	116198341	MED13L	AS	A/G	0.62	0.81	0.10	5.7 × 10^‒16^	0.78	0.42	0.68	5.4 × 10^‒1^
12	rs10858966	90567026	ATP2B1	E	C/G	0.29	0.26	0.03	9.2 × 10^‒15^	0.02	0.86	2.17	6.9 × 10^‒1^
12	rs2024385	12888438	APOLD1	E	A/T	0.42	−0.26	0.03	5.9 × 10^‒18^	0.46	−0.57	0.59	3.4 × 10^‒1^
12	rs11571376	1059556	RAD52	E	C/G	0.70	−0.18	0.03	5.7 × 10^‒8^	0.73	−0.49	0.71	4.9 × 10^‒1^
12	rs6487543	26438189	SSPN	E	A/G	0.77	0.30	0.05	6.3 × 10^‒10^	0.08	−0.41	1.05	7.0 × 10^‒1^
**12**	**rs2681492**	**90013089**	**ATP2B1**	**E/AS/AA**	**G/A**	**0.17**	**−0.97**	**0.16**	**5.8 × 10^‒8^**	**0.15**	**1.84**	**0.79**	**2.1 × 10^‒2^**
12	rs10850411	115387796	TBX5‐TBX3	E	T/C	0.70	0.35	—	5.4 × 10^‒8^	0.61	−0.45	0.60	4.5 × 10^‒1^
**12**	**rs17249754**	**90060586**	**ATP2B1**	**E**	**G/A**	**0.84**	**0.93**	—	**1.8 × 10^‒18^**	**0.85**	**−1.58**	**0.80**	**4.8 × 10^‒2^**
12	rs10437954	58003922	ARHGEF25	E	A/G	0.90	−0.41	0.05	1.6 × 10^‒14^	0.67	0.61	0.66	3.5 × 10^‒1^
12	rs5742643	102837863	IGF1	E	A/G	0.25	−0.22	0.03	2.0 × 10^‒10^	0.26	−0.67	0.69	3.3 × 10^‒1^
12	rs7963801	79685226	SYT1	E	T/C	0.41	−0.24	0.03	2.8 × 10^‒14^	0.01	3.33	2.54	1.9 × 10^‒1^
12	rs7976167	24210599	SOX5	E	T/C	0.69	0.18	0.03	3.8 × 10^‒8^	0.84	1.61	0.82	5.1 × 10^‒2^
13	rs9532243	32191408	RXFP2	E	A/C	0.48	0.22	0.03	8.2 × 10^‒14^	0.55	−0.33	0.59	5.8 × 10^‒1^
13	rs606950	22298923	FGF9	E	A/G	0.62	0.27	0.03	3.2 × 10^‒18^	0.47	0.88	0.60	1.4 × 10^‒1^
13	rs78474310	73826901	KLF5	E	A/G	0.96	−0.47	0.07	1.5 × 10^‒10^	0.99	−0.72	3.38	8.3 × 10^‒1^
13	rs9526707	51489186	RNASEH2B	E	A/G	0.32	−0.20	0.03	2.7 × 10^‒10^	0.12	−1.59	0.91	8.0 × 10^‒2^
13	rs9549328	113636156	MCF2L	E	T/C	0.23	0.32	0.06	1.5 × 10^‒8^	0.15	0.59	0.89	5.1 × 10^‒1^
14	rs8014182	103859962	MARK3	E	T/C	0.14	−0.33	0.04	5.2 × 10^‒14^	0.02	0.19	1.93	9.2 × 10^‒1^
14	rs11159091	75074316	LTBP2	E	A/G	0.46	0.20	0.03	6.7 × 10^‒11^	0.01	2.00	2.77	4.7 × 10^‒1^
14	rs11623535	72462381	RGS6	E	A/G	0.74	0.21	0.03	1.0 × 10^‒9^	0.57	0.71	0.60	2.4 × 10^‒1^
14	rs17115145	30122409	PRKD1	E	T/C	0.40	0.18	0.03	7.4 × 10^‒9^	0.57	−0.33	0.58	5.7 × 10^‒1^
14	rs9888615	53377540	FERMT2	E	T/C	0.29	−0.32	0.05	3.5 × 10^‒10^	0.62	0.38	0.63	5.4 × 10^‒1^
14	rs8016306	63928546	PPP2R5E	E	A/G	0.80	0.34	0.06	3.7 × 10^‒9^	0.15	−0.10	0.84	9.1 × 10^‒1^
15	rs1563894	68635775	TGA11	E	A/G	0.19	−0.09	—	2.9 × 10^‒8^	0.69	0.17	0.63	7.8 × 10^‒1^
15	rs2521501	91437388	FURIN‐FES	E	T/A	0.31	0.65	—	5.2 × 10^‒9^	0.21	−0.39	0.76	6.1 × 10^‒1^
15	rs1378942	75077367	CYP1A1‐ULK3	E	C/A	0.35	0.61	—	5.7 × 10^‒23^	0.97	−0.03	1.70	9.9 × 10^‒1^
15	rs35199222	81013037	ABHD17C	E	A/G	0.45	0.32	0.05	5.2 × 10^‒12^	0.07	0.39	1.18	7.5 × 10^‒1^
15	rs11632436	86295286	RP11−158M2.4	E	C/G	0.50	0.22	0.03	2.0 × 10^‒13^	0.21	−0.31	0.73	6.7 × 10^‒1^
15	rs3743157	85680532	PDE8A	E	A/C	0.17	0.29	0.04	4.2 × 10^‒13^	0.71	0.10	0.66	8.8 × 10^‒1^
15	rs4965529	100145224	MEF2A	E	T/G	0.17	−0.26	0.04	5.4 × 10^‒11^	0.31	−0.16	0.64	8.0 × 10^‒1^
16	rs11639856	24788645	TNRC6A	E/AA	A/T	0.19	−0.34	0.06	1.3 × 10^‒8^	0.19	0.90	0.78	2.5 × 10^‒1^
16	rs11643209	75331044	CFDP1	E	T/G	0.42	−0.34	0.05	1.8 × 10^‒12^	0.74	0.08	0.70	9.1 × 10^‒1^
16	rs7187540	85318302	LINC00311	E	A/C	0.34	−0.20	0.03	1.0 × 10^‒8^	0.15	0.01	0.89	9.9 × 10^‒1^
17	rs4925159	18185510	TOP3A	E	A/G	0.43	0.22	0.03	9.7 × 10^‒13^	0.73	0.29	0.63	6.5 × 10^‒1^
17	rs34430710	56876627	PPM1E	E	A/T	0.68	−0.21	0.03	5.0 × 10^‒11^	0.89	0.95	0.89	2.9 × 10^‒1^
17	rs1036902	58950791	BCAS3	E	T/C	0.84	−0.25	0.04	1.7 × 10^‒9^	0.19	−0.14	0.73	8.5 × 10^‒1^
17	rs1551355	30032420	RP11−805L22.1	E	T/C	0.23	0.21	0.04	3.9 × 10^‒9^	0.03	−0.05	1.94	9.8 × 10^‒1^
17	rs12940887	47402807	ZNF652	E	T/C	0.38	0.36	—	1.8 × 10^‒10^	0.07	−0.48	1.43	7.4 × 10^‒1^
17	rs57927100	75317300	SEPT9	E	C/G	0.26	−0.31	0.05	4.0 × 10^‒14^	0.78	−0.60	0.70	3.9 × 10^‒1^
17	rs2467099	73949045	ACOX1	E	T/C	0.22	−0.30	0.06	3.3 × 10^‒8^	0.12	−0.82	0.95	3.9 × 10^‒1^
17	rs12941318	1333598	CRK	E	T/C	0.49	−0.27	0.05	2.5 × 10^‒8^	0.75	0.67	0.66	3.1 × 10^‒1^
17	rs12946454	43208121	PLCD3	E	T/A	0.28	0.50	0.17	1.0 × 10^‒8^	0.06	−0.06	1.20	9.6 × 10^‒1^
17	rs7406910	46688256	HOXB7	E/AS	T/C	0.12	−0.46	—	3.8 × 10^‒8^	0.26	0.38	0.65	5.6 × 10^‒1^
17	rs112280096	79367409	RP11−1055B8.6	E	A/C	0.36	−0.20	0.04	1.3 × 10^‒9^	0.06	0.68	1.45	6.4 × 10^‒1^
18	rs12958173	42141977	SETBP1	E	A/C	0.31	0.36	0.05	1.4 × 10^‒13^	0.32	0.72	0.63	2.6 × 10^‒1^
18	rs12454712	60845884	BCL2	E	T/C	0.62	0.19	0.03	5.8 × 10^‒9^	0.76	0.86	0.70	2.2 × 10^‒1^
18	rs10048404	54578482	WDR7	E	T/C	0.37	−0.26	0.03	1.9 × 10^‒16^	0.05	−1.12	1.33	4.9 × 10^‒1^
18	rs11876341	48799991	MEX3C	E	A/G	0.69	−0.21	0.03	1.8 × 10^‒10^	0.94	2.01	1.48	1.8 × 10^‒1^
19	rs4247374	7252756	INSR	E	T/C	0.14	−0.59	0.08	1.2 × 10^‒18^	0.02	−0.93	2.23	6.7 × 10^‒1^
20	rs1327235	10969030	JAG1	E	G/A	0.46	0.34	—	1.9 × 10^‒8^	0.59	−0.03	0.59	9.6 × 10^‒1^
20	rs6015450	57751117	GNAS‐EDN3	E	G/A	0.12	0.90	—	3.9 × 10^‒23^	0.18	0.51	0.74	5.0 × 10^‒1^
21	rs12627651	44760603	CRYAA‐SIK1	E	A/G	0.29	0.39	0.05	2.6 × 10^‒14^	0.07	1.70	1.28	5.9 × 10^‒1^
22	rs4823006	29451671	ZNRF3	E/AA	G/A	0.42	−0.26	0.05	7.9 × 10^‒9^	0.40	0.86	0.58	1.4 × 10^‒1^
22	rs28578714	50727921	PLXNB2	E	T/C	0.61	0.21	0.03	2.5 × 10^‒10^	0.53	−0.59	0.58	3.1 × 10^‒1^

Note

Bold indicate *p* < 5.0 × 10^−2^ for replication analysis.

Abbreviations: AA, African ancestry; AS, Asian ancestry; Chr, chromosomes; E, European ancestry; EA, effect allele; EAF, effect allele frequency; RA, reference allele; SE, standard error; SNP, single nucleotide polymorphism; β, Effect size estimates correspond to mean difference in mmHg per effect allele for systolic or diastolic blood pressure, adjusted for age and body mass index.

a Given with respect to Build 37 (GRCh37/hg19).

b Adjusted for age and body mass index.

c Indicate same β direction in both the discovery and EMaBS populations.

**Table 3:** Loci associated with diastolic blood pressure identified from previous GWAS and results of replication in Entebbe Mother and Baby Study (EMaBS) sample

Chr	SNP	Position^a^	Nearest gene	Population	EA/RA	Discovery sample				EMaBS sample			
						EAF	β	*SE*	*p*‐value	EAF	β^b^	*SE*	*p*‐value
1	rs17367504	11862778	MTHFR/NPPB	E	G/A	0.15	−0.55	—	3.5 × 10^‒19^	0.09	−0.53	0.97	5.8 × 10^‒1^
**1**	**rs2169137**	**204497913**	**MDM4**	**E/AS/AA**	**G/C**	**0.27**	**−0.36**	**0.07**	**5.9 × 10^‒8^**	**0.23**	**1.32**	**0.61**	**3.1 × 10^‒2^**
1	rs13306561	11865804	MTHFR	E/AS/AA	G/A	0.15	−0.52	0.09	3.0 × 10^‒19^	0.27	0.04	0.58	9.4 × 10^‒1^
1	rs2932538	113216543	MOV10	E	G/A	0.75	0.24	—	9.9 × 10^‒10^	0.85	−0.25	0.70	3.6 × 10^‒1^
1	rs4846049	11850365	MTHFR‐NPPB	E	T/G	0.33	−0.55	0.10	6.7 × 10^‒8^	0.51	0.72	0.50	1.6 × 10^‒1^
1	rs6686889	25030470	chr1mb25	E	T/C	0.25	0.19	0.03	3.6 × 10^‒9^	0.36	−0.28	0.53	5.9 × 10^‒1^
1	s12405515	172357441	DNM3	E	T/G	0.56	−0.17	0.03	1.4 × 10^‒9^	0.20	0.68	0.66	3.0 × 10^‒1^
1	s12408022	217718789	GPATCH2	E	T/C	0.26	0.20	0.03	2.4 × 10^‒10^	0.06	−0.43	1.14	7.1 × 10^‒1^
1	rs10916082	227252626	CDC42BPA	E	A/G	0.73	−0.18	0.03	8.4 × 10^‒9^	0.52	−0.06	0.52	9.1 × 10^‒1^
1	rs2760061	228191075	WNT3A	E	A/T	0.47	0.23	0.03	2.1 × 10^‒16^	0.62	−0.02	0.56	9.7 × 10^‒1^
1	rs953492	243471192	SDCCAG8	E	A/G	0.46	0.22	0.03	7.4 × 10^‒16^	0.68	−0.18	0.56	7.5 × 10^‒1^
1	rs2004776	230848702	AGT	E	T/C	0.23	0.32	0.06	5.0 × 10^‒8^	0.52	−0.45	0.52	3.9 × 10^‒1^
1	rs1565716	29549216	MECR	E	A/G	0.07	0.21	0.03	3.5 × 10^‒10^	0.09	0.66	1.02	5.2 × 10^‒1^
1	rs35981664	218549354	TGFB2	E	A/T	0.69	−0.16	0.02	2.0 × 10^‒17^	0.99	−0.58	3.69	8.8 × 10^‒1^
1	rs12142296	46541679	PIK3R3	E	T/G	0.86	−0.16	0.03	8.9 × 10^‒11^	0.98	1.09	1.66	5.1 × 10^‒1^
1	rs72704264	145713305	CD160	E	C/G	0.21	0.12	0.02	3.6 × 10^‒8^	0.03	0.58	1.44	6.9 × 10^‒1^
2	rs1446468	164963486	FIGN‐GRB14	E	T/C	0.53	−0.50	0.07	6.9 × 10^‒9^	0.95	−0.61	1.17	6.1 × 10^‒1^
2	rs16823124	183224127	PDE1A	E	A/G	0.42	0.26	0.04	2.0 × 10^‒10^	0.11	−0.06	0.80	9.4 × 10^‒1^
**2**	**rs55701159**	**25139596**	**ADCY3**	**E**	**T/G**	**0.89**	**0.29**	**0.04**	**7.2 × 10^‒11^**	**0.89**	**−1.98**	**0.79**	**1.3 × 10^‒2^**
2	rs4952611	40567743	SLC8A1	E	T/C	0.58	−0.16	0.03	4.0 × 10^‒8^	0.72	−0.08	0.59	9.0 × 10^‒1^
2	rs2579519	96675166	GPAT2‐FAHD2CP	E	T/C	0.63	−0.20	0.03	4.8 × 10^‒12^	0.72	0.01	0.61	9.9 × 10^‒1^
2	rs7592578	191439591	TMEM194B	E	T/G	0.19	−0.24	0.04	9.5 × 10^‒12^	0.27	−0.81	0.61	1.8 × 10^‒1^
2	rs1063281	218668732	TNS1	E	T/C	0.60	−0.20	0.03	1.3 × 10^‒12^	0.59	0.28	0.55	6.1 × 10^‒1^
2	rs1975487	55809054	PNPT1	E	A/G	0.46	−0.16	0.03	1.8 × 10^‒9^	0.68	0.02	0.55	9.7 × 10^‒1^
2	rs1220128	158499902	ACVR1C	E	C/G	0.85	0.19	0.02	6.2 × 10^‒15^	0.40	0.20	0.53	7.1 × 10^‒1^
2	rs1996992	219651349	CYP27A1	E	T/G	0.05	−0.30	004	4.7 × 10^‒14^	0.09	−1.00	0.93	2.8 × 10^‒1^
2	rs13001283	127183454	GYPC	E	A/G	0.16	0.15	0.02	1.9 × 10^‒10^	0.11	0.81	0.79	3.1 × 10^‒1^
2	rs7606205	144146311	ARHGAP15	E	A/C	0.70	−0.13	0.02	2.4 × 10^‒11^	0.49	−0.40	0.53	4.5 × 10^‒1^
2	rs34570306	146272860	ZEB2	E	T/C	0.53	−0.12	0.02	1.2 × 10^‒11^	0.07	−0.00	1.04	1.0 × 10^‒1^
2	rs4851462	98357163	ZAP70	E	T/C	0.63	−0.12	0.02	4.0 × 10^‒11^	0.90	−0.46	0.91	6.2 × 10^‒1^
2	rs2707238	38094149	LINC00211	E	C/G	0.28	0.10	0.02	6.8 × 10^‒8^	0.28	−0.42	0.60	4.8 × 10^‒1^
3	rs11128722	14958126	FGD5	E	A/G	0.56	−0.17	0.03	5.1 × 10^‒10^	0.32	0.55	0.53	3.0 × 10^‒1^
3	rs918466	64710253	ADAMTS9	E	A/G	0.41	−0.18	0.03	1.7 × 10^‒11^	0.90	0.57	0.86	5.1 × 10^‒1^
3	rs36022378	49913705	CAMKV‐ACTBP13	E	T/C	0.80	−0.20	0.03	4.7 × 10^‒9^	0.98	−0.37	2.44	8.8 × 10^‒1^
3	rs743757	50476378	CACNA2D2	E	C/G	0.14	0.25	0.04	2.4 × 10^‒10^	0.55	−0.60	0.54	2.7 × 10^‒1^
3	s9827472	56726646	FAM208A	E	T/C	0.37	−0.18	0.03	4.3 × 10^‒10^	0.48	0.19	0.52	7.0 × 10^‒1^
3	rs2306374	138119952	MRAS	E	T/C	0.84	−0.18	0.03	7.4 × 10^‒9^	0.96	2.03	1.37	1.4 × 10^‒1^
3	rs12374077	185317674	SENP2	E	C/G	0.35	0.16	0.03	9.2 × 10^‒9^	0.47	0.52	0.52	3.2 × 10^‒1^
3	rs143112823	154707967	RP11−439C8.2	E	A/G	0.09	−0.40	0.05	1.4 × 10^‒14^	0.09	−0.41	0.99	6.8 × 10^‒1^
3	rs419076	169100886	MECOM	E	T/C	0.47	0.24	—	2.1 × 10^‒12^	0.57	−0.29	0.54	5.8 × 10^‒1^
3	rs319690	47927484	MAP4	E	T/C	0.50	0.28	0.05	1.8 × 10^‒8^	0.44	0.26	0.55	6.3 × 10^‒1^
3	rs1706003	194299967	TMEM44	E	T/G	0.47	0.12	0.02	5.8 × 10^‒12^	0.01	−3.51	2.17	1.1 × 10^‒1^
3	rs11923667	101268080	TRMT10C	E	A/T	0.41	0.12	0.02	3.1 × 10^‒11^	0.27	−1.06	0.57	6.5 × 10^‒2^
3	rs6777317	197070959	DLG1	E	A/G	0.92	0.12	0.02	1.5 × 10^‒10^	0.90	0.76	0.92	4.1 × 10^‒1^
3	rs4634143	23163749	UBE2E2	E	T/C	0.30	0.12	0.02	7.9 × 10^‒10^	0.07	0.10	1.06	9.3 × 10^‒1^
3	rs3774372	41877414	ULK4	E	T/C	0.83	−0.37	—	9.0 × 10^‒14^	0.77	−0.72	0.59	2.2 × 10^‒1^
4	rs13139571	156645513	GUCY1A3‐GUCY1B3	E	C/A	0.76	0.26	—	2.2 × 10^‒10^	0.86	0.89	0.75	2.4 × 10^‒1^
4	rs6825911	111381638	ENPEP	AS	C/T	0.51	0.39	0.07	9.0 × 10^‒9^	0.61	0.46	0.55	4.0 × 10^‒1^
4	rs2291435	38387395	TBC1D1‐FLJ13197	E/AA	T/C	0.52	−0.16	0.03	4.3 × 10^‒9^	0.30	0.07	5.58	9.0 × 10^‒1^
4	rs66887589	120509279	PDE5A	E	T/C	0.52	−0.22	0.03	3.4 × 10^‒15^	0.62	−0.46	0.55	4.0 × 10^‒1^
4	rs1458038	81164723	FGF5	E	T/C	0.29	0.46	—	8.5 × 10^‒25^	0.04	0.98	1.35	4.7 × 10^‒1^
4	rs223361	103769304	UBE2D3	E	T/C	0.66	0.17	0.02	2.7 × 10^‒20^	0.48	−0.10	0.54	8.5 × 10^‒1^
4	rs28667801	26785356	STIM2	E	A/T	0.59	−0.16	0.02	1.9 × 10^‒19^	0.87	−0.01	0.78	9.9 × 10^‒1^
4	rs55829085	2165493	POLN	E	A/C	0.95	−0.28	0.04	3.0 × 10^‒11^	0.99	2.13	2.50	4.0 × 10^‒1^
**4**	**rs7694000**	**95324968**	**PDLIM5**	**E**	**A/T**	**0.54**	**−0.10**	**0.02**	**3.5 × 10^‒8^**	**0.21**	**2.03**	**0.63**	**1.3 × 10^‒3^**
4	rs62312401	116987529	NDST4‐TRAM1L1	AA	C/G	0.94	1.13	0.24	3.5 × 10^‒9^	0.04	−2.14	1.39	1.3 × 10^‒1^
5	rs12521868	131784393	C5orf56	E	T/G	0.37	−0.19	—	6.1 × 10^‒11^	0.04	−0.06	1.44	9.6 × 10^‒1^
5	rs1173771	32815028	NPR3‐C5orf23	E	G/A	0.60	0.26	—	9.1 × 10^‒12^	0.82	1.04	0.72	1.5 × 10^‒1^
5	rs11953630	157845402	EBF1	E	T/C	0.37	−0.28	—	3.8 × 10^‒13^	0.13	1.27	0.77	9.9 × 10^‒2^
5	rs10077885	114390121	TRIM36	E	A/C	0.50	−0.17	0.03	4.0 × 10^‒11^	0.65	−0.40	0.55	4.7 × 10^‒1^
5	rs6891344	123136656	CSNK1G3	E	A/G	0.82	0.23	0.03	1.6 × 10^‒11^	0.72	0.45	0.56	4.1 × 10^‒1^
5	rs10078021	75038431	POC5	E	T/G	0.63	−0.16	0.03	1.3 × 10^‒8^	0.15	−0.41	0.69	5.5 × 10^‒1^
5	rs72812846	173377636	CPEB4	E	A/T	0.28	−0.21	0.03	2.2 × 10^‒11^	0.04	−0.18	1.43	9.0 × 10^‒1^
5	rs10062049	61553881	KIF2A	E	T/C	0.14	0.22	0.02	4.5 × 10^‒18^	0.32	0.62	0.63	3.2 × 10^‒1^
**5**	**rs954767**	**3706050**	**IRX1**	**E**	**A/C**	**0.74**	**−0.15**	**0.02**	**4.2 × 10^‒14^**	**0.68**	**1.14**	**0.54**	**3.6 × 10^‒2^**
5	rs55747751	132397351	HSPA4	E	A/G	0.08	−0.22	0.03	1.4 × 10^‒11^	0.01	−0.17	2.43	9.4 × 10^‒1^
**5**	**rs4286632**	**66291370**	**MAST4**	**E**	**A/G**	**0.73**	**0.12**	**0.02**	**1.9 × 10^‒9^**	**0.84**	**1.50** c	**0.74**	**4.3 × 10^‒2^**
5	rs2188962	131770805	C5orf56	E/AA	T/C	0.37	−0.20	0.03	3.0 × 10^‒11^	0.03	1.25	1.70	4.6 × 10^‒1^
5	rs12515541	57095011	ACTBL2	E	T/G	0.61	0.12	0.02	6.2 × 10^‒11^	0.49	0.08	0.54	8.9 × 10^‒1^
6	rs926552	29548089	SNORD32B	E/AA	T/C	0.11	−0.26	0.05	7.2 × 10^‒8^	0.21	−0.25	0.63	7.0 × 10^‒1^
6	rs10943605	79655477	PHIP	E/AA	A/G	0.46	0.16	0.03	3.3 × 10^‒9^	0.28	0.10	0.57	8.6 × 10^‒1^
6	rs13205180	51832494	PKHD1	E	T/C	0.49	0.17	0.03	7.0 × 10^‒10^	0.12	0.43	0.81	6.0 × 10^‒1^
6	rs9372498	118572486	SLC35F1	E	A/T	0.08	0.33	0.05	1.8 × 10^‒11^	0.10	0.11	0.87	9.0 × 10^‒1^
6	rs147212971	166178451	PDE10A	E	T/C	0.06	−0.36	0.06	1.6 × 10^‒9^	0.17	0.34	0.74	6.5 × 10^‒1^
6	rs1799945	26091179	HFE	E	G/C	0.14	0.46	—	1.5 × 10^‒15^	0.02	−0.43	1.90	8.2 × 10^‒1^
**6**	**rs805303**	**31616366**	**BAT2‐BAT5**	**E**	**G/A**	**0.61**	**0.23**	**—**	**3.0 × 10^‒11^**	**0.40**	**1.75** c	**0.54**	**1.0 × 10^‒3^**
6	rs13209747	127115454	RSPO3	E/AA/AS	T/C	0.19	0.56	0.12	2.4 × 10^‒11^	0.07	−0.50	0.96	6.0 × 10^‒1^
6	rs17080102	151004770	PLEKHG1	E/AA/AS	C/G	0.10	−0.74	0.15	1.9 × 10^‒11^	0.15	−1.21	0.70	8.8 × 10^‒2^
6	rs9472135	43809802	VEGFA	E	T/C	0.70	0.15	0.02	4.3 × 10^‒16^	0.77	0.58	0.62	3.5 × 10^‒1^
6	rs668459	139835689	CITED2	E	T/C	0.59	−0.11	0.02	1.0 × 10^‒10^	0.28	0.02	0.57	9.7 × 10^‒1^
6	rs598682	154418759	OPRM1	E	A/G	0.25	−0.11	0.02	7.2 × 10^‒8^	0.03	0.23	1.31	8.6 × 10^‒1^
**7**	**rs17428471**	**27337867**	**EVX1‐HOXA**	**E/AA/AS**	**T/G**	**0.14**	**0.61**	**0.14**	**1.6 × 10^‒9^**	**0.13**	**2.01** c	**0.79**	**1.1 × 10^‒2^**
7	rs2969070	2512545	CHST12‐LFNG	E	A/G	0.63	−0.18	0.03	2.9 × 10^‒11^	0.97	−0.24	1.58	8.8 × 10^‒1^
**7**	**rs11556924**	**129663496**	**ZC3HC1**	**E**	**T/C**	**0.38**	**−0.21**	**0.03**	**8.2 × 10^‒15^**	**0.01**	**6.90**	**2.72**	**1.2 × 10^‒2^**
7	rs6969780	27159136	HOXA3	E/AA	C/G	0.13	0.26	0.05	1.1 × 10^‒8^	0.39	0.68	0.54	2.1 × 10^‒1^
7	rs891511	150704843	NOS3	E/AA	A/G	0.37	−0.26	0.03	2.0 × 10^‒16^	0.59	−0.55	0.52	3.0 × 10^‒1^
7	rs1947228	96461649	SHFM1	E	T/C	0.42	−0.14	0.02	2.6 × 10^‒16^	0.96	1.01	1.29	4.4 × 10^‒1^
7	rs1722886	134215259	AKR1B10	E	A/T	0.57	0.12	0.02	3.8 × 10^‒12^	0.29	−0.05	0.57	9.3 × 10^‒1^
7	rs9638084	156311745	LINC01006	E	A/G	0.40	0.12	0.02	8.5 × 10^‒11^	0.55	−0.40	0.51	4.3 × 10^‒1^
7	rs11563582	27351650	EVX1‐HOXA	AA	A/G	0.13	1.02	0.17	8.4 × 10^‒10^	0.17	0.80	0.71	2.6 × 10^‒1^
8	rs78192203	142375073	GPR20	AA	T/A	0.80	0.77	0.14	1.3 × 10^‒8^	0.79	−0.29	0.65	6.5 × 10^‒1^
8	rs2978098	101676675	SNX31	E	A/C	0.54	0.17	0.03	1.5 × 10^‒9^	0.03	−0.02	1.70	9.9 × 10^‒1^
8	rs62524579	144060955	P11‒273G15.2	E	A/G	0.53	−0.18	0.03	3.8 × 10^‒9^	0.37	0.28	0.55	6.2 × 10^‒1^
8	rs10087782	141858620	PTK2	E	T/C	0.45	0.13	0.02	3.0 × 10^‒14^	0.82	0.32	0.67	6.3 × 10^‒1^
8	rs1047030	22428708	SORBS3	E	A/G	0.81	0.13	0.02	5.7 × 10^‒8^	0.96	−0.80	1.23	5.2 × 10^‒1^
9	rs4364717	21801530	MTAP	E	A/G	0.55	−0.18	0.03	1.3 × 10^‒10^	0.24	0.33	0.61	5.8 × 10^‒1^
9	rs76452347	35906471	HRCT1	E/AA	T/C	0.19	−0.23	0.04	6.8 × 10^‒10^	0.09	−0.16	0.92	8.6 × 10^‒1^
9	rs7020564	109670016	ZNF462	E	A/C	0.70	−0.11	0.02	6.7 × 10^‒9^	0.12	−0.54	0.82	5.1 × 10^‒1^
10	rs4746172	75855842	VCL	E	C/T	—	0.23	0.04	9.1 × 10^‒8^	0.23	−0.49	0.66	4.6 × 10^‒1^
10	rs1801253	115805056	ADRB1	E	G/C	0.27	−0.36	0.06	9.5 × 10^‒10^	0.34	−0.45	0.56	4.2 × 10^‒1^
10	rs10995311	64564934	ADO	E/AA	G/C	0.38	−0.20	0.03	2.1 × 10^‒11^	0.03	1.36	1.47	3.6 × 10^‒1^
10	rs1530440	63524591	C10orf107	E	T/C	0.19	−0.39	0.06	1.0 × 10^‒9^	0.03	−0.87	1.58	5.8 × 10^‒1^
10	rs4590817	63467553	C10orf107	E	G/C	0.84	0.42	—	1.3 × 10^‒12^	0.84	−0.81	0.68	2.4 × 10^‒1^
10	rs2782980	115781527	ADRB1	E	T/C	0.20	−0.28	0.05	9.6 × 10^‒8^	0.47	−0.76	0.53	1.5 × 10^‒1^
10	rs1813353	18707448	CACNB2	E	T/C	0.68	0.42	—	2.3 × 10^‒15^	0.84	0.16	0.73	8.3 × 10^‒1^
10	rs603424	102075479	PKD2L1	E	A/G	0.18	0.18	0.02	1.2 × 10^‒14^	0.75	0.36	0.63	5.7 × 10^‒1^
10	rs10906391	13523937	BEND7	E	T/C	0.32	0.13	0.02	7.6 × 10^‒12^	0.03	−0.27	1.65	8.6 × 10^‒1^
10	rs4373814	18419972	CACNB2(5′)	E	G/C	0.55	−0.22	—	4.4 × 10^‒10^	0.39	−0.35	0.53	5.1 × 10^‒1^
10	rs11191548	104846178	CYP17A1/NT5C2	E	T/C	0.91	0.46	—	9.4 × 10^‒13^	0.98	1.23	1.42	3.9 × 10^‒1^
11	rs4601790	65353906	EHBP1L1	E/AS	G/A	0.27	−0.02	0.04	9.9 × 10^‒9^	0.06	−0.32	1.05	7.6 × 10^‒1^
11	rs7103648	47461783	RAPSN‐PSMC3‐SLC39A13	E	A/G	0.61	−0.24	0.03	9.0 × 10^‒19^	0.85	0.78	0.74	3.0 × 10^‒1^
**11**	**rs751984**	**61278246**	**LRRC10B**	**E**	**T/C**	**0.88**	**0.38**	**0.04**	**4.2 × 10^‒20^**	**0.79**	**1.56** c	**0.63**	**1.4 × 10^‒2^**
11	rs900145	13293905	ARNTL	E/AA	G/A	0.34	−0.20	0.03	1.8 × 10^‒8^	0.52	0.97	0.54	7.0 × 10^‒2^
11	rs11030119	27728102	BDNF	E	A/G	0.31	−0.16	0.03	2.9 × 10^‒8^	0.35	−0.36	0.54	5.1 × 10^‒1^
11	rs67330701	69079707	MYEOV	E	T/C	0.09	−0.37	0.05	2.1 × 10^‒12^	0.01	0.92	2.27	6.9 × 10^‒1^
**11**	**rs633185**	**100593538**	**FLJ32810‐TMEM133**	**E**	**G/C**	**0.28**	**−0.33**	—	**2.0 × 10^‒15^**	**0.23**	**−1.49** c	**6.23**	**1.7 × 10^‒2^**
11	rs381815	16902268	PLEKHA7	E	T/C	0.26	0.35	—	5.3 × 10^‒14^	0.26	0.58	0.62	3.4 × 10^‒1^
11	rs1401454	16250183	SOX6	E/AA/AS	T/C	0.46	0.45	0.10	5.1 × 10^‒10^	0.46	−0.27	0.52	6.1 × 10^‒1^
11	rs360153	9762274	SWAP70	E	T/C	0.42	−0.22	0.02	4.4 × 10^‒36^	0.52	−0.30	0.52	5.7 × 10^‒1^
11	rs11026586	22515533	RP11−34N19.1	E	A/G	0.07	0.29	0.03	2.7 × 10^‒17^	0.06	0.43	1.21	7.3 × 10^‒1^
11	rs875106	70005641	ANO1	E	A/G	0.52	−0.13	0.02	1.7 × 10^‒14^	0.71	1.02	5.8	7.7 × 10^‒2^
11	rs4420291	74374950	POLD3	E	A/G	0.51	0.10	0.02	2.2 × 10^‒8^	0.33	0.08	0.58	8.9 × 10^‒1^
**11**	**rs7129220**	**10350538**	**ADM**	**E**	**G/A**	**0.89**	**−0.30**	—	**6.4 × 10^‒8^**	**0.92**	**−0.79** c	**1.01**	**4.4 × 10^‒3^**
12	rs17249754	90060586	ATP2B1	E	G/A	0.84	0.52	—	1.2 × 10^‒18^	0.85	−1.89	0.69	7.0 × 10^‒1^
12	rs10850411	115387796	TBX5/TBX3	E	T/C	0.70	0.25	—	5.4 × 10^‒10^	0.61	0.32	0.53	5.5 × 10^‒1^
12	rs1060105	123806219	SBNO1	E/AS	T/C	0.21	−0.18	—	3.1 × 10^‒8^	0.05	−1.08	1.22	3.8 × 10^‒1^
12	rs2384550	115352731	TBX5‐TBX3	E	A/G	0.35	−0.35	0.06	3.7 × 10^‒8^	0.35	−0.93	0.53	7.9 × 10^‒2^
12	rs35444	115552437	TBX3	AS	A/G	0.75	0.50	0.05	1.3 × 10^‒10^	0.56	−0.66	0.53	2.1 × 10^‒1^
12	rs7302981	50537815	CERS5	E/AA	A/G	0.34	0.25	0.03	9.4 × 10^‒19^	0.10	0.90	0.84	2.8 × 10^‒1^
12	rs7132012	8832203	RP11−20D14.4	E	A/G	0.68	0.15	0.02	3.2 × 10^‒17^	0.57	0.08	0.51	8.8 × 10^‒1^
12	rs1271309	124820705	NCOR2	E	A/G	0.16	−0.20	0.02	1.5 × 10^‒16^	0.08	0.22	0.92	8.1 × 10^‒1^
12	rs7137749	57098040	NACA	E	T/C	0.37	0.14	0.02	7.2 × 10^‒15^	0.52	−0.44	0.52	4.1 × 10^‒1^
12	rs7134060	96717095	CDK17	E	A/G	0.45	−0.11	0.02	1.1 × 10^‒9^	0.26	−0.93	0.60	1.2 × 10^‒1^
12	rs75507123	5417856	RP11−1038A11.3	E	T/G	0.13	−0.14	0.03	3.9 × 10^‒8^	0.02	−0.66	1.70	7.0 × 10^‒1^
12	rs1098708	27321112	STK38L	E	A/G	0.28	−0.10	0.02	4.6 × 10^‒8^	0.82	1.08	0.67	1.1 × 10^‒1^
13	rs55684003	97988689	MBNL2	E	A/G	0.70	0.12	0.02	1.0 × 10^‒10^	0.95	−1.83	1.14	1.1 × 10^‒1^
13	rs9563529	58316637	PCDH17	E	T/G	0.21	0.12	0.02	1.4 × 10^‒8^	0.26	0.56	0.59	3.4 × 10^‒1^
14	rs11628933	60700903	PPM1A	E	C/G	0.23	−0.12	0.02	3.1 × 10^‒9^	0.40	−0.41	0.54	4.5 × 10^‒1^
14	rs4424827	35110857	SNX6	E	T/C	0.57	−0.10	0.02	2.1 × 10^‒8^	0.94	−0.38	7.20	9.6 × 10^‒1^
**15**	**rs7178615**	**66869072**	**RP11‒321F6.1**	**E**	**A/G**	**0.37**	**−0.18**	**0.03**	**2.6 × 10^‒10^**	**0.19**	**1.42**	**0.69**	**4.1 × 10^‒2^**
15	rs62012628	79070000	ADAMTS7	E	T/C	0.29	−0.24	0.03	5.1 × 10^‒12^	0.31	−0.03	0.58	9.6 × 10^‒1^
15	rs12906962	95312071	chr15mb95	E	T/C	0.68	−0.22	0.03	5.6 × 10^‒14^	0.21	−0.73	0.64	2.5 × 10^‒1^
**15**	**rs1378942**	**75077367**	**CYP1A1‐ULK3**	**E**	**C/A**	**0.36**	**0.48**	**0.09**	**6.0 × 10^‒8^**	**0.97**	**2.99** c	**1.48**	**4.4 × 10^‒2^**
15	rs2521501	91437388	FURIN‐FES	E	T/A	0.31	0.36	—	1.9 × 10^‒15^	0.21	−1.11	0.66	9.5 × 10^‒2^
15	rs873122	92702020	SLCO3A1	E	C/G	0.72	0.12	0.02	6.5 × 10^‒10^	0.94	2.00	1.16	8.5 × 10^‒2^
15	rs7180952	85162551	ZSCAN2	E	T/C	0.54	−0.10	0.02	9.8 × 10^‒9^	0.75	0.02	0.61	9.8 × 10^‒1^
**15**	**rs62004794**	**68454523**	**PIAS1**	**E**	**A/G**	**0.44**	**−0.10**	**0.02**	**3.4 × 10^‒8^**	**0.60**	**−1.16** c	**0.55**	**3.6 × 10^‒2^**
16	rs12921187	4943019	PPL	E	T/G	0.43	−0.17	0.03	2.5 × 10^‒10^	0.96	−2.36	1.23	5.5 × 10^‒2^
16	rs72799341	30936743	FBXL19	E	A/G	0.24	0.19	0.03	5.8 × 10^‒9^	0.09	0.03	0.90	9.8 × 10^‒1^
16	rs8059962	81574197	CMIP	E	T/C	0.42	−0.17	0.03	1.3 × 10^‒9^	0.61	0.37	0.52	4.8 × 10^‒1^
16	rs1126464	89704365	DPEPI	E/AA	C/G	0.22	0.24	0.03	2.4 × 10^‒13^	0.06	−0.90	1.10	4.2 × 10^‒1^
16	rs45474499	66914492	PDP2	E	T/C	0.05	0.36	0.04	8.5 × 10^‒18^	0.07	0.85	1.09	4.3 × 10^‒1^
16	rs7185555	69131281	HAS3	E	C/G	0.15	−0.15	0.02	2.3 × 10^‒10^	0.31	0.42	0.56	4.6 × 10^‒1^
**16**	**rs9932866**	**706067**	**WDR90**	**E**	**A/G**	**0.37**	**0.12**	**0.02**	**2.9 × 10^‒10^**	**0.90**	**−1.61**	**0.80**	**4.5 × 10^‒2^**
17	rs4308	61559625	ACE	E	A/G	0.37	0.21	0.03	6.8 × 10^‒14^	0.03	−1.29	1.73	4.5 × 10^‒1^
17	rs12940887	47402807	ZNF652	E	T/C	0.38	0.27	—	2.3 × 10^‒14^	0.07	0.41	1.25	7.4 × 10^‒1^
18	rs12958173	42141977	SETBP1	E	A/C	0.31	0.18	0.03	5.8 × 10^‒10^	0.31	0.68	0.56	2.2 × 10^‒1^
18	rs745821	48142854	MAPK4	E	T/G	0.76	0.19	0.03	1.4 × 10^‒9^	0.70	0.21	0.58	7.1 × 10^‒1^
18	rs34163044	51851616	STARD6	E	A/C	0.42	0.15	0.02	9.6 × 10^‒17^	0.31	−0.13	0.59	8.3 × 10^‒1^
18	rs11665020	10879503	PIEZO2	E	C/G	0.32	−0.14	0.02	2.8 × 10^‒14^	0.14	−0.18	0.72	8.1 × 10^‒1^
18	rs4800420	20158965	CTAGE1	E	A/G	0.29	0.12	0.02	5.2 × 10^‒10^	0.17	−0.63	0.70	3.7 × 10^‒1^
19	rs167479	11526765	RGL3	E/AA	T/G	0.45	−0.30	0.03	4.2 × 10^‒28^	0.17	−0.23	0.71	7.4 × 10^‒1^
19	rs62104477	30294991	CCNE1	E	T/G	0.33	0.18	0.03	1.2 × 10^‒9^	0.25	0.47	0.61	4.4 × 10^‒1^
19	rs4247374	7252756	INSR	E	T/C	0.14	−0.39	0.03	2.1 × 10^‒22^	0.02	−1.85	1.96	3.5 × 10^‒1^
19	rs2304130	19789528	ZNF101	E/AS	A/G	0.91	−0.29	—	2.0 × 10^‒8^	0.74	−0.63	0.59	2.9 × 10^‒1^
19	rs9710247	40760449	AKT2	E	A/G	0.45	0.16	0.03	1.6 × 10^‒9^	0.90	−1.29	0.92	1.6 × 10^‒1^
19	rs1821295	32590773	AC011518.1	E	T/C	0.70	−0.14	0.02	3.1 × 10^‒13^	0.92	−0.06	1.14	9.6 × 10^−1^
20	rs6095241	47308798	PREX1	E/AS	A/G	0.45	−0.17	—	4.8 × 10^−9^	0.60	−0.42	0.53	4.4 × 10^−1^
20	rs6108168	8626271	PLCB1	E	A/C	0.25	−0.21	0.03	1.1 × 10^−11^	0.62	−0.08	0.53	8.8 × 10^−1^
20	rs1327235	10969030	JAG1	E	G/A	0.46	0.30	—	1.4 × 10^−15^	0.59	−0.09	0.52	8.6 × 10^−1^
20	rs6015450	57751117	GNAS‐EDN3	E	G/A	0.12	0.56	—	5.6 × 10^−23^	0.18	0.74	0.65	2.6 × 10^−1^
20	rs1232482	11886643	BTBD3	E	T/C	0.40	−0.12	0.02	6.1 × 10^−12^	0.18	−1.26	0.70	7.3 × 10^−2^
**21**	**rs12627651**	**44760603**	**CRYAA‐SIK1**	**E**	**A/G**	**0.29**	**0.20**	**0.03**	**1.4 × 10^−11^**	**0.07**	**2.55** c	**1.12**	**2.3 × 10^−2^**

Note

Bold indicate *p* < 5.0 × 10^−2^ for replication analysis.

Abbreviations: AA, African ancestry; AS, Asian ancestry; Chr, chromosomes; E, European ancestry; EA, effect allele; EAF, effect allele frequency; RA, reference allele; SE, standard error;SNP, single nucleotide polymorphism; β, Effect size estimates correspond to mean difference in mmHg per effect allele for systolic or diastolic blood pressure, adjusted for age and body mass index.

a Given with respect to Build 37 (GRCh37/hg19).

b Adjusted for age and body mass index.

c Indicate same β direction in both the discovery and EMaBS populations.

Of the 30 SNPs previously known to be associated with BP specifically in individuals of African origin, three (*ATP2B* rs2681492, *MDM4* rs2169137 and *EVX1*/*HOXA* rs17428471) were associated with BP in the present study. Only the *EVX1*/*HOXA* rs17428471 had the same effect direction as in the discovery population. The *BAT2*/*BAT5* rs805303 variant was associated with systolic BP and diastolic BP of the 40 SNPs tested for association with both traits. The G allele of the *BAT2*/*BAT5* rs805303 variant was associated with higher systolic and higher diastolic BP among adolescents in this study: the same effect direction observed in the discovery population for both traits.

There were 370 independent tests (197 for systolic BP, 173 for diastolic BP) conducted, thus approximately 19 SNPs (370 × 0.05 = 18.5) would be expected to be associated with BP at *p* < .05 by chance alone. None of the replicated SNPs met a Bonferroni corrected significance threshold (0.05/370 = 1.35 × 10^−4^), although one (rs6712094 intergenic between *FIGN* and *GRB14*) was very close (*p* = 2.6 × 10^−4^) for association with systolic BP. The most strongly associated SNPs for association with diastolic BP were rs805303 between *BAT2* and *BAT5* (*p* = 1.0 × 10^−3^) and *PDLIM5* rs7694000 (*p* = 1.3 × 10^−3^).

## 4 DISCUSSION

To our knowledge, this is the first genetic analysis examining variants associated with BP among African adolescents. We hypothesized that common genetic variants (unique or not unique to populations in Africa) were associated with systolic and, or diastolic BP in Ugandan adolescents and that these associations may overlap with associated variants identified in previous studies of Africans. The GWAS revealed no novel or previously identified variant associated with systolic or diastolic BP in our study population. Thirty‐three SNPs were associated with BP in the replication analysis, with the direction of effect consistent with the discovery population for 14 SNPs. There were no SNPs reaching a Bonferroni‐adjusted significance level. None of the replicated SNPs were located in genes with monogenic effect on hypertension (Ehret & Caulfield, 2013).

The SNPs most strongly associated with either systolic or diastolic BP were of borderline significance and none have been reported as associated with BP in previous BP GWASs. The most strongly associated SNPs were mostly common variants with modest effect sizes and might uniquely influence BP in African population. It is important for larger genetic studies of African population to investigate the role of these SNPs in BP regulation among Africans. These top SNPs are potential candidates for replication analysis in African populations.

The failure to identify variants strongly associated with BP presumably occurred because the study was underpowered to detect effects of rare variants or small effects of common variants. Blood pressure is most likely a polygenic trait influenced by the simultaneous presence of several gene variants each with a small effect size and contributing in an additive manner to BP expression. Thus, the large effect sizes that this study had good power to detect, may not be realistic. For example, the present study had 80% power to detect a 3.2 mmHg change in mean systolic BP for a minor allele frequency of 20% at genome‐wide significance level, *p* < 5 × 10^−8^. Many of the variants reliably associated with BP in adults have an effect size of 0.5 mmHg or less (Evangelou et al., 2018). In addition to the limitation caused by the relatively small sample size, imputation did not allow inference for rare variants not included in the imputation SNP panel.

Few GWAS “top SNPs” from non‐African populations have been replicated in populations of African ancestry (Adeyemo et al., 2009; Franceschini et al., 2013; Kayima et al., 2017). Variants associated with BP in populations of African origin might be different from variants that influence BP in Caucasian populations or not in LD with the BP causing variants. Our replication study was limited to variants associated with BP from previous GWAS of BP in other populations. Thirty‐three SNPs identified from previous BP GWAS were replicated, most of these were previously identified in populations of non‐African origins. Of the identified loci, *PAX2* is essential in the development of the renal epithelium (Dressler & Woolf, 1999) and plays a critical role in kidney development (Hou, Chen, & Wang, 2011). The kidneys are critical in BP regulation. Two of the replicated SNPs are located on *ATP2B1*. *ATP2B1* is involved in calcium homeostasis (Hirawa, Fujiwara, & Umemura, 2013). The *ATP2B1* rs2681492, *MDM4* rs2169137, *EVX1*/*HOXA* rs17428471 SNPs previously associated with BP in transethnic populations (African, Caucasian and Asian) were associated with adolescent BP in the present study. These genes most likely influence BP across different ethnic groups.

Replication studies in diverse populations have returned mixed results. This current study conducted 370 tests using 330 SNPs, of which 33 SNPs (one SNP for both traits) were associated with BP. Failure to replicate most variants associated with BP in other populations could be due to differences in minor allele frequencies across populations or differences in LD patterns combined with a poor understanding of the causative variants or due to spurious initial findings. Blood pressure is likely to be influenced by the simultaneous presence of several genetic variants, each conferring a small change in BP.

Although none of the variants reached Bonferroni level of significance more associated variants were identified than expected under the null suggesting that some of these variants found to be associated could be worthy of further follow‐up. Fourteen variants more than those expected by chance (19 variants) under the null hypothesis were associated with BP in this study.

Of the 40 SNPs tested for association with both traits, only *BAT2*/*BAT5* rs805303 was associated with both traits in the adolescents, suggesting that not many genetic loci have influence on both systolic and diastolic BP. Similar to an earlier replication study among adult Ugandans (Kayima et al., 2017), the *ATP2B1* rs2681492 was associated with BP in these Ugandan adolescents, but with an opposite effect direction to the discovery population (Hoffmann et al., 2017) and Ugandan adults (Kayima et al., 2017). The G allele of the *ATP2B1* rs2681492 was associated with lower systolic BP in Ugandans adults (Kayima et al., 2017) but with higher systolic BP in the present study.

Some loci may have varying roles in BP regulation across different populations. A European study investigated SNPs associated with BP at different age epochs (using independent samples for each age group): prepuberty (age 4–7 years), pubertal (8–12 years), and postpubertal (13–20 years). The A allele of *TGA11* rs1563894 was associated with lower systolic BP in prepuberty while the T allele of *SMARCA2*/*VLDLR* rs872256 was associated with higher systolic BP during puberty (Parmar et al., 2016). No SNP was associated with BP in the postpubertal period, and no SNP was consistently associated with BP across all three age groups. The *TGA11* rs1563894 and *SMARCA2*/*VLDLR* rs872256 were not replicated in this present study.

The present study has several strengths. This is the first BP GWAS of an African population and the first candidate gene analysis among adolescents residing in Africa. Participants in this study were similar to nonparticipants with respect to most baseline characterizes. Rigorous quality control procedures were used during the measurement of the various variables including BP and genotyping. Data from this study can contribute to future BP GWAS meta‐analyses. Key limitations of this study were that it was underpowered to detect effects of rare variants and to enable testing for effect modification by sex and other environmental variables, and the lack of a replication sample from a similar setting with which to confirm our GWAS findings.

Future work should take advantage of various African cohorts to form consortia that can enable the conduct of GWAS meta‐analysis well powered to identify rare and low‐frequency variants that may be associated with BP in African populations. Future candidate gene analysis using a sample from a different geographical region or ethnic background should investigate for interactions between variants, this might help our understanding of the etiology of BP. It is possible that multiple interacting variants (rare and common) are influencing BP levels in this population. Although we did not formally allow for multiple testing in the replication analysis, the current study had 33 associated SNPs 14 more than expected by chance. Polygenic scores analysis of variants associated with BP among African populations may explain the missing BP heritability.

In summary, we conducted the first genetic study of BP phenotypes among Ugandan adolescents. Although this study did not identify novel BP variants, replication of some previously identified variants suggests that some genetic variants may universally influence BP susceptibility. Large scale studies in African populations are required to identify novel and evaluate previously reported loci.

## CONFLICT OF INTEREST

None declared.

## Data Availability

The data that support the findings of this study will be available through the European Genome‐Phenome Archive (EGA) upon lifting of the embargo for the VaccGene paper.
