# Supplementary material for: A genome‐wide association and replication study of blood pressure in Ugandan early adolescents
Source: Mol Genet Genomic Med. 2019 Aug 30;7(10):e00950. doi: 10.1002/mgg3.950 (PMC6785527; doi:10.1002/mgg3.950)
Supplement: Supplementary file 1 [file MGG3-7-e00950-s001.docx]

| **Table S1:** | | | **Characteristics of the Entebbe Mother and Baby Study participants included and not included in the genetic analysis study (N=2345)** | | | | | |
| --- | --- | --- | --- | --- | --- | --- | --- | --- |
| **Mothers’ characteristics during pregnancy** | | | | **Participated**  **(n=815)** | | | **Did not participate (n=1530)** | |
|  |  |  |  | **Number** | | **Percentage/Mean (SD)** | **Number** | **Percentage/Mean (SD)** |
|  | Age (years)^*^ | | | | 815 | 24.5 (5.6) | 1530 | 23.3 (5.2) |
|  | Parity (number of children) ^*^ | | | | 815 | 3.0 (1.8) | 1530 | 2.7 (1.7) |
|  | Body mass index^*^ | | | | 809 | 24.0 (3.2) | 1509 | 24.1 (3.2) |
|  | Household SES index (1 lowest, 6 highest) | | | | 806 | 3.8 (1.2) | 1495 | 3.6 (1.2) |
|  | Education level | | | |  |  |  |  |
|  |  | None | | | 20 | 2.5 | 66 | 4.3 |
|  |  | Primary | | | 392 | 48.2 | 800 | 52.4 |
|  |  | Senior | | | 317 | 39.0 | 554 | 36.3 |
|  |  | Tertiary | | | 84 | 10.3 | 108 | 7.1 |
|  | Marital status | | | |  |  |  |  |
|  |  | Single | | | 85 | 10.4 | 222 | 14.5 |
|  |  | Married/cohabiting | | | 709 | 87.1 | 1257 | 82.2 |
|  |  | Separated/widowed | | | 20 | 2.5 | 51 | 3.3 |
|  | Area of residence | | | |  |  |  |  |
|  |  | Urban | | | 574 | 71.3 | 1027 | 68.1 |
|  |  | Rural | | | 231 | 28.7 | 481 | 31.9 |
|  | Infections | | | |  |  |  |  |
|  |  | Asymptomatic malaria | | | 75 | 9.4 | 173 | 11.5 |
|  |  | Schistosomiasis | | | 134 | 16.5 | 287 | 18.8 |
|  |  | Hookworm | | | 322 | 39.6 | 703 | 46.2 |
|  |  | Ascaris | | | 21 | 2.6 | 33 | 2.2 |
|  |  | Trichuris | | | 67 | 8.2 | 139 | 9.1 |
|  |  | Trichostrongylus | | | 8 | 1.0 | 14 | 0.9 |
| **Participants’ characteristics** | | | | |  |  |  |  |
|  | Birthweight (Kg)^*^ | | | | 692 | 3.2 (0.5) | 1204 | 3.2 (0.5) |
|  | Sex | | | |  |  |  |  |
|  |  | Male | | | 417 | 51.1 | 794 | 52.0 |
|  | Birth type | | | |  |  |  |  |
|  |  | Singleton | | | 802 | 98.4 | 1488 | 97.3 |
|  |  | Multiple | | | 13 | 1.6 | 42 | 2.8 |
|  | Feeding at 6 weeks of age | | | |  |  |  |  |
|  |  | Exclusive breastfeeding | | | 579 | 71.5 | 893 | 63.6 |
|  |  | Mixed feeding | | | 219 | 27.0 | 502 | 35.8 |
|  |  | Weaned | | | 12 | 1.8 | 9 | 0.6 |
|  | HIV status | | | |  |  |  |  |
|  |  | Unexposed | | | 731 | 89.7 | 1334 | 87.2 |
|  |  | Exposed not infected | | | 76 | 9.3 | 150 | 9.8 |
|  |  | Infected | | | 8 | 1.0 | 46 | 3.0 |
|  | Place of birth | | | |  |  |  |  |
|  |  | Entebbe Hospital | | | 611 | 75.0 | 1069 | 70.3 |
|  |  | Home | | | 85 | 10.4 | 179 | 11.8 |
|  |  | Others | | | 119 | 14.6 | 273 | 18.0 |
|  | Mode of delivery | | | |  |  |  |  |
|  |  | Normal | | | 731 | 89.7 | 1385 | 91.0 |
|  |  | Caesarean section | | | 77 | 9.5 | 120 | 7.9 |
|  |  | Instrumentation | | | 7 | 0.9 | 17 | 1.2 |
| ^†^ Mean value with standard deviation (SD) presented.  Percentages may total to ± 100 due to rounding.  Household socioeconomic status (SES) was a composite variable and could take values 1 (low) to 6 (high)  Missing data for (a) mother’s characteristics: body mass index 27; household SES 44; education 4; marital status 1; place of residence 32; asymptomatic malaria 43; schistosomiasis 9; hookworm 9; ascaris 9; trichuris 9; trichostrongylus 9; (b) child’s characteristics: sex 2; birth weight 449; feeding status 131; place of delivery 9; mode of delivery 8. | | | | | | | | |
